# Agricultural intensification reduces selection of putative plant growth-promoting rhizobacteria in wheat

**DOI:** 10.1093/ismejo/wrae131

**Published:** 2024-07-11

**Authors:** Tessa E Reid, Vanessa N Kavamura, Adriana Torres-Ballesteros, Monique E Smith, Maïder Abadie, Mark Pawlett, Ian M Clark, Jim A Harris, Tim H Mauchline

**Affiliations:** Sustainable Soils and Crops, Rothamsted Research, Harpenden, Hertfordshire AL5 2JQ, United Kingdom; School of Water, Energy and Environment, Cranfield University, Cranfield, Bedfordshire MK43 0AL, United Kingdom; Sustainable Soils and Crops, Rothamsted Research, Harpenden, Hertfordshire AL5 2JQ, United Kingdom; Sustainable Soils and Crops, Rothamsted Research, Harpenden, Hertfordshire AL5 2JQ, United Kingdom; Sustainable Soils and Crops, Rothamsted Research, Harpenden, Hertfordshire AL5 2JQ, United Kingdom; Department of Ecology, Swedish University of Agricultural Sciences, Uppsala SE-750 07, Sweden; Sustainable Soils and Crops, Rothamsted Research, Harpenden, Hertfordshire AL5 2JQ, United Kingdom; Present address: INRAE, UR1264 MycSA, CS2032, 33882 Villenave d’Ornon, France; School of Water, Energy and Environment, Cranfield University, Cranfield, Bedfordshire MK43 0AL, United Kingdom; Sustainable Soils and Crops, Rothamsted Research, Harpenden, Hertfordshire AL5 2JQ, United Kingdom; School of Water, Energy and Environment, Cranfield University, Cranfield, Bedfordshire MK43 0AL, United Kingdom; Sustainable Soils and Crops, Rothamsted Research, Harpenden, Hertfordshire AL5 2JQ, United Kingdom

**Keywords:** plant growth-promoting rhizobacteria, PGPR, wheat, fertilization, ploidy, culture-independent, culture-dependent, Bacteroidota

## Abstract

The complex evolutionary history of wheat has shaped its associated root microbial community. However, consideration of impacts from agricultural intensification has been limited. This study investigated how endogenous (genome polyploidization) and exogenous (introduction of chemical fertilizers) factors have shaped beneficial rhizobacterial selection. We combined culture-independent and -dependent methods to analyze rhizobacterial community composition and its associated functions at the root–soil interface from a range of ancestral and modern wheat genotypes, grown with and without the addition of chemical fertilizer. In controlled pot experiments, fertilization and soil compartment (rhizosphere, rhizoplane) were the dominant factors shaping rhizobacterial community composition, whereas the expansion of the wheat genome from diploid to allopolyploid caused the next greatest variation. Rhizoplane-derived culturable bacterial collections tested for plant growth-promoting (PGP) traits revealed that fertilization reduced the abundance of putative plant growth-promoting rhizobacteria in allopolyploid wheats but not in wild wheat progenitors. Taxonomic classification of these isolates showed that these differences were largely driven by reduced selection of beneficial root bacteria representative of the Bacteroidota phylum in allopolyploid wheats. Furthermore, the complexity of supported beneficial bacterial populations in hexaploid wheats was greatly reduced in comparison to diploid wild wheats. We therefore propose that the selection of root-associated bacterial genera with PGP functions may be impaired by crop domestication in a fertilizer-dependent manner, a potentially crucial finding to direct future plant breeding programs to improve crop production systems in a changing environment.

## Introduction

The transformation of natural habitats to agricultural systems greatly increased food production [1] but was accompanied by reduced crop genetic diversity [2] by selecting high-yielding dwarf crops [3] which are reliant on unsustainable levels of inorganic fertilizers [4]. Agricultural intensification, the combination of plant domestication, agrochemical inputs, mechanization, and irrigation, was a crucial accomplishment in human history providing a continuous food supply for societal prosperity. However, the longevity of our current agricultural system has recently been tested through political [5], societal [6], and climatic [7] disturbances, leading to economic crises, which have highlighted the need for self-sufficient, climate positive food production systems.

Plants coexist with a large diversity of microorganisms and share a complex coevolutionary history [8]. This relationship developed naturally when plants colonized land ~450 million years ago allowing soil microbes such as bacteria to form key mutualistic relationships with plants. This resulted in the soil environment surrounding the plant root, the rhizosphere [9], becoming a zone of maximum microbial activity, different to that of the surrounding soil, where key transactions are made between root and bacteria essential for the health of each species. The rhizosphere compartment was later divided to allow for greater differentiation between soil around the roots to the root surfaces themselves, defining soil on the root surface to which microbes directly adhere as the rhizoplane [10]. The term “plant growth-promoting rhizobacteria” or “PGPR” was later defined by Kloepper and his colleagues [11] to further distinguish rhizobacteria, bacteria competent in colonizing the root environment, based on their ability to perform functions that promote the growth and health of the plant in a symbiotic relationship by three key mechanisms: (i) nutrient acquisition, (ii) disease suppression, and (iii) abiotic stress tolerance [12]. Since then, the potential of PGPR in sustainable agriculture and methods to study them have been well established [12]. Cereal domestication dating back to around 10 000 years ago, however, focused on optimizing aerial parts of the plant, particularly seeds, for human consumption without contemplation of belowground plant-microbe interactions.

Bread wheat (*Triticum aestivum*), one of the world’s leading sources of food, is an allopolyploid (6× = AABBDD = 42) composed of the genomes of three different species (summarized in Fig. S1). Over a relatively brief period of evolutionary time, wheat domestication and polyploidy significantly altered plant phenotype. Additionally, during the Green Revolution, selection for mutant alleles of the *Reduced height* (*Rht*) dwarfing genes in modern wheat cultivars resulted in short, high-yielding wheat plants, which, when combined with agrochemical management and optimal conditions, increased yields without productivity losses caused by lodging [13]. The well-documented domestication history and close relatedness of wild and domesticated wheat provide a unique framework for comparative analyses of plant-associated microbial communities. Moreover, it allows us to address the selection of associated microbial communities under cultivation.

Increasing evidence suggests that the complex evolutionary history of wheat (genome hybridizations [14] and domestication events [15, 16]) has shaped the associated root microbial community [17–30]. However, the impact of fertilization, which defined the Green Revolution [31], has only received limited consideration. Here, we used 16S rRNA gene amplicon sequencing, rhizobacterial isolations, functional bioassay analysis to identify putative PGPR (bacteria with PGP traits) (Fig. S2), and taxonomic classification of these putative PGPR vs. non-PGPR (Fig. S3) bacterial communities to investigate the possible impacts of plant breeding on selection of beneficial rhizobacteria in an agricultural soil under contrasting fertilization conditions. We analyzed the rhizobacterial communities associated with 17 *Triticum* as well as 2 wild grass *Aegilops* accessions (Table S1) representing three plant genetic groups (diploid, tetraploid, and hexaploid) along a transect of wheat domestication and breeding. Our goal was to incorporate the use of bioassays to test the function of culturable bacteria at the root–soil interface. We targeted PGP traits related to nutrient acquisition via solubilization mechanisms and siderophore production using well established bioassays previously used to isolate PGPR (Fig. S2) and assess impacts of crop domestication and chemical fertilizer application on root associated microbiome structure and PGP functions.

## Materials and methods

### Experimental design

*Triticum* accessions were selected to represent three plant genetic groups at three time points along an evolutionary transect of wheat domestication and breeding: two diploid accessions (*Triticum urartu* and *Triticum monococcum,* wild relatives from which wheat was domesticated), four tetraploid accessions (*Triticum dicoccoides*, *Triticum carthlicum, Triticum polonicum*, and *Triticum turanicum*), and two hexaploid accessions (*Triticum spelta* and *Triticum macha*). These seeds were kindly provided by the Germplasm Resources Unit (a national capability supported by the BBSRC). Nine *T. aestivum* cultivars were also selected to represent modern, domesticated bread wheat [32, 33]. Additionally, two wild grass species (*Aegilops tauschii* and *Aegilops speltoides*), which are thought to be genome derivatives for *T. aestivum*, were also selected (Table S1, Fig. S1).

Field soil was collected from Woburn, Stackyard bare-fallow soil mine (latitude: 52°00′04.3"N, 0°36′49.0"W), a well-draining sandy loam soil from the Rothamsted Research experimental farm at Woburn, Bedfordshire (UK). The soil is a Cottenham series [34] classified as a Cambric Arenosol (FAO) and has been maintained as a bare fallow for over 50 years. It was chosen to reduce the legacy effect of prior cropping systems. Topsoil (0–20 cm) was collected in August 2019 across three replicate plots and thoroughly mixed to produce a homogenized soil. Soil was sieved (5-mm mesh), air dried, and stored at 4°C in polythene bags prior to use.

Seeds were surface sterilized and pregerminated (Supplementary Notes: Method S1) before being transplanted to individual wells on a seed tray (1× seedling per well) and grown in a glasshouse at Rothamsted Research for 2 weeks (20°C, 16 h/day light regime, watered daily) before vernalization for 12 weeks (4°C; 8-h light and 16-h dark). Plants were then transferred to 9 × 9 × 10 cm pots filled with 500-g soil (1× plant per pot) with and without NPK granules (15% N, 9% P, 11% K, 2% Mg with micro-nutrients (B, Cu, Fe, Mn, Mo, Zn); Osmocote, UK) (2.5 g per pot) in a two-factor randomized block design with cultivar and fertilizer as factors (Fig. S4). As a control (referred to in text and figures as unplanted soil), plant-free pots were established and given the same fertilizer treatments as pots with plants. Overall, 19 accessions were grown in one soil, plus an unplanted control (20 conditions) with and without fertilizer (2 conditions) in quadruplicates (160 pots).

### Sample collection, DNA extraction, PCR amplification, and amplicon sequencing

At the start of flowering (Zadoks growth stage 61) [35] (Fig. S5), soil samples were collected (Supplementary Notes: Method S2). Roots were vigorously shaken in a bag to release tightly attached soil (i.e. rhizosphere), mixed to homogenize and split between two 5-ml screw cap vials. One vial was flash frozen in liquid nitrogen and stored at −80°C for culture-independent work, and one vial was stored at 4°C for culture work. The root system was excised and placed in a sterile 50-ml centrifuge tube for subsequent rhizoplane work. To obtain rhizoplane soil, collected root samples were weighed and 0.9-ml sterile distilled water was added for every 0.1 g of root. Samples were shaken vigorously for 10 min using an orbital shaker. For culture-dependent work: 1-ml soil solution was transferred to a 2-ml cryo-tube, 1-ml glycerol (50%; autoclaved) was added, tubes were vortexed, and flash frozen in liquid nitrogen before being stored at −80°C. For culture-independent work: 4-ml soil solution was centrifuged (2 min, RT, 15 000 rpm), supernatant discarded, and remaining soil was flash frozen in liquid nitrogen and stored at −80°C.

Genomic DNA was extracted from unplanted, rhizosphere, and rhizoplane soil (~0.25 g) using the DNeasy PowerSoil Pro kit (Qiagen, Venlo, Netherlands) (Supplementary Notes: Method S3). For PCR amplification, the universal 314F and 806R PCR primers with barcodes applied were used to amplify the V3-V4 region of bacterial 16S rRNA genes. Amplicon preparation followed a previously published protocol [36] (Supplementary Notes: Method S3). After amplification, PCR products were purified and pooled. Finally, the amplicon library was sequenced with MiSeq v3 chemistry 300 base paired-end sequencing (Illumina, San Diego, CA, United States). Following sequencing: demultiplexing, merging reads, and taxonomy assignment using the SILVA138 database were performed using the QIIME2 pipeline (Supplementary Notes: Method S4).

### Culture-dependent functional analysis for plant growth-promoting traits

The ability of rhizoplane-colonizing bacteria to solubilize casein, phosphate, potassium, iron, and zinc was tested using previously established bioassays, as previously described [37]. We chose to only assess the rhizoplane as our previous work clearly demonstrated a more pronounced treatment effect in the rhizoplane vs. rhizosphere microbial isolates [37]. To obtain a library of rhizoplane-colonizing bacteria, rhizoplane soil previously frozen in glycerol was thawed, vortexed, serially diluted, spread on tryptone soya agar (TSA) (Tryptone Soya Broth (TSB; 1/10th concentration) (Oxoid, Basingstoke, UK) and Bacto Agar) (5 replicate plates per sample), and incubated (25°C, 4 days). Colony counts were measured using ImageJ [38, 39]. Individual colonies were picked and inoculated in 150 μl TSB (1/10th concentration) in 96-well plates and incubated (25°C, 2 days) prior to functional analysis; two wells were left uninoculated as negative media controls. A sterile 96-prong inoculating manifold was used to spot individual inoculated isolates from the 96-well plate liquid cultures onto each bioassay before 150-μl glycerol (50%; autoclaved) was added to liquid cultures and stored at −80°C. Assays were incubated (25°C, 5–7 days); positive isolates were counted per sample for each functional assay (Fig. S2). In total, 14 288 rhizobacterial isolates were isolated from 145 rhizoplane soil samples and 8 unplanted soil samples (94 isolates per sample) and tested for nutrient-solubilizing traits.

To test the impact of polyploidization and fertilization on the proportion of positive isolates, negative binomial generalized linear models (glm) were applied to the dataset using the glm function in R v4.2.2 [40] using RStudio [41] followed by analysis of deviance (*P* < .05) tests (anova() function with Chisq test); the predictmeans package [42] was used to make pairwise comparisons based on fertilization and genome interaction (*P* < .05). Absolute abundances were calculated from colony counts pertaining to colony forming units per gram of soil (CFU g/soil) and normalized by logarithmic transformation. All graphs were created in GraphPad Prism version 10.0.2 (171) for MacOS (GraphPad Software, Boston, MA, USA). This software was also used to calculate two-way analysis of variance (ANOVA) and pair-wise *t*-tests with Šidák correction [43] for absolute bacterial abundance and absolute abundance of isolates with PGP traits. See Supplementary Notes: Method S5 for full details of statistical analyses.

### PGPR vs. non-PGPR amplicon sequencing

To representatively test a proportion of the culture-dependent microbiome, samples from statistical blocks 1 to 3 (Fig. S4) were sequenced by 16S rRNA gene amplicon sequencing. This amounted to 11 092 isolates from 118 samples from three plant genetic groups and an unplanted control, with and without the addition of fertilizer. Previously frozen glycerol stocks of bacterial isolates in 96-well plates were used to inoculate fresh TSB (1/10th conc.; 150 μl) in 96-well plates and incubated (25°C, 2 days). Samples were then taken by pipetting 100-μl culture from selected wells using custom scripts designed on the Opentrons Protocol designer on the OT-2 robot (Opentrons, Long Island City, NY, USA). From the 118 × 96-well plates, isolates previously characterized with PGP traits (“PGPR”) and isolates identified with no PGP traits (“non-PGPR”) were sorted into 15-ml tubes (Fig. S3). Cultures were vortexed before 2 ml was aliquoted into tubes (Eppendorf), pelleted, and the supernatant removed. Bacterial pellets were subjected to GenElute Bacterial Genomic DNA extraction (Sigma-Aldrich, St. Louis, MO, USA) using the lysozyme utilizing Gram-positive bacterial preparation method according to the manufacturer’s instructions. DNA samples were sent to Novogene (UK) Company Limited (Milton, Cambridge, UK) and sequenced on the NovoSeq PE250 sequencing platform (Illumina) using primers 341F and 806R to amplify the V3-V4 region of bacterial 16S rRNA genes. Sequences were processed the same as culture-independent sequences (Supplementary Notes: Method S4).

### Microbial community analysis

All analyses were performed using R v4.2.2 [40] in RStudio [41] using the Phyloseq [44] (v1.34.0) package. Sequencing of the culture-independent 16S rRNA gene amplicon library resulted in 10 298 ASVs and 5.3 million reads in 286 samples and sequencing of the culture-dependent isolate 16S rRNA gene amplicon library resulted in 6440 ASVs and 1.8 million reads in 196 samples. In the culture-independent amplicon dataset, ASVs unclassified at phylum level (63) and samples where only one species was observed in alpha rarefaction (*n* = 27) were removed from downstream analysis. The function isContaminant() from the decontam (v1.16.0) package [45] was used to filter contaminant ASVs present in negative controls of DNA extraction and sequencing from the datasets (93 ASVs and 14 ASVs from culture-independent and culture-dependent datasets, respectively). Finally, ASVs were filtered using a custom function whereby only ASVs present in three out of the four replicates for each group (soil × fertilization × plant) for the culture-independent dataset and two out of the three replicates for each group (plant × fertilization × isolate_function) for the culture-dependent dataset were kept. The resulting Phyloseq objects were used for all further analyses.

Bacterial alpha diversity indices pertaining to richness (observed species), evenness (Simpson), diversity (Shannon) (R package: microbiome [46]), and relatedness (Faith’s Phylogenetic Diversity (PD)) (R package: picante v1.8.2 [47]) were calculated based on the rarefied ASV table (2000 reads). The alpha diversity rarefaction plots created using the MicrobiotaProcess [48] package confirmed that a sufficient depth of coverage was achieved at this cut-off (Fig. S6). Box plot figures were created using ggboxplot() in the ggpubr [49] package. The global significance of differences in bacterial diversity among fertilization, soil type, and wheat ploidy level and pairwise multiple comparisons between groups was tested using a type III two-way ANOVA (R package: car [50] v3.1–2) on rank transformed data to correct for normality [51] followed by pairwise comparisons which were conducted on the estimated marginal means for the factor combinations using the emmeans [52] package.

For beta diversity analysis, ASV counts were normalized by the variance stabilizing transformation method implemented in DESeq2 v1.36.0 [53]. Bray–Curtis distance was calculated using ordinate(); Principal Coordinate Analysis (PCoA) and Canonical Analysis of Principal coordinates (CAP) were conducted using plot_ordination(), both from the Phyloseq package. PERMANOVA (permutational analysis of variance) tests were performed using adonis2() (R package: vegan [54]) with 9999 permutations and block factor (based on the randomized block experimental setup).

Differential abundance analyses were performed using the DESeq2 v1.36.0 [53] package. The resulting data were used to produce volcano plots with ggplot2 v.3.4.2 [55] and ternary plots using the methods reported in a previous study [56].

Phylum level community composition of culture-dependent data was investigated by relative abundance of culture-dependent ASV counts. The percentage of phyla in the rhizoplane soil of wild wheat progenitors (AA, BB, DD) and allopolyploid wheats (AABB, AABBDD) as well as unplanted soils, treated with and without fertilizer, were used to calculate the absolute abundance of each phylum in groups based on CFU per gram of soil as described above. Phylum-level bar plots were created using Prism10.

For full details on all statistical analysis performed, see Supplementary Notes: Method S5.

## Results

### Fertilization is the dominant factor shaping wheat rhizobacterial communities

**Figure 1:**
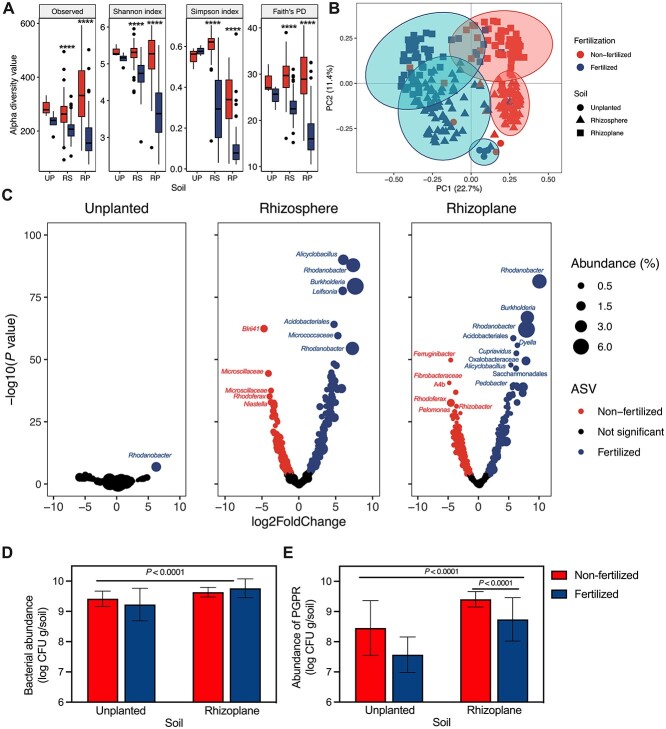
Fertilization effects on wheat rhizobacterial communities; (A) alpha diversity estimates at ASV level for unplanted (UP), rhizosphere (RS), and rhizoplane (RP) bacterial communities in non-fertilized and fertilized wheat; with median (line) and hinges at first and third quartiles (25th and 75th percentiles), and significant differences as determined by two-way ANOVA (type III) tests followed by pairwise interactions with Tukey correction are depicted: “^*^^*^^*^^*^” for *P* < .0001, between fertilization groups; (B) PCoA plot of bacterial composition based on Bray–Curtis distance for bacterial communities at ASV level; the percentage shown in each axis corresponds to the proportion of variation explained, and (C) volcano plots for all bacterial ASVs found in unplanted (406 ASVs from 7 samples), rhizosphere (1265 ASVs from 127 samples), and rhizoplane (1319 ASVs from 110 samples) soil samples; the *x*-axis represents the abundance fold change on log2 scale, and the *y*-axis represents the negative log10 of the adjusted *P*-value as calculated by DESeq2 differential abundance analysis; red points indicate ASVs with an adjusted *P*-value < .0001 that are differentially more abundant in samples from non-fertilized wheat; blue points indicate ASVs with an adjusted *P*-value < .0001 that are differentially more abundant in samples from fertilized wheat, and points in black indicate ASVs with an adjusted *P*-value > .0001; a full list of ASVs can be found in Supplementary Data S3; (D) absolute abundance (log CFU g/soil; CFU is colony-forming units) of culturable rhizobacteria isolated from soil samples on 10% TSA; (E) absolute abundance (log CFU g/soil) of nutrient solubilizing rhizobacteria as determined by functional bioassays, and values at the top indicate the *P*-values of the two-way ANOVA between soils and the adjusted *P-*value for pairwise interactions with Šidák correction between fertilization groups.

The effects of fertilization on the total rhizobacterial community across all wheat species were assessed. We used a low nutrient soil (Table S2) to emphasize the effects of chemical fertilizer addition, demonstrated by the higher aerial plant biomass and ear length in fertilized soils (Fig. S7A and B; Supplementary Notes: Result S1). Alpha diversity metrices were significantly lower in the rhizosphere and rhizoplane of fertilized wheat, whereas there were no statistical differences between treatments in unplanted soil (Fig. 1A, Data S1). PCoA showed clustering of samples by fertilization along the first principal coordinate axis (22.7% variance) and by soil niche along the second principal coordinate axis (11.4% variance) (Fig. 1B). Multifactorial PERMANOVA indicated that fertilization and soil compartment, as well as their interaction, significantly shaped wheat rhizobacterial communities but that ploidy, ancestral class, genome, and plant species also significantly contributed significantly to the total variance (Data S2). Wheat clearly shapes its associated rhizobacterial community under both fertilization conditions, with 42.5% and 55.9% of ASVs showing differential abundance in the rhizosphere and rhizoplane, respectively, in contrast to unplanted soils, where 0.2% of ASVs were differentially more abundant (Fig. 1C, Data S3).

Rhizoplane bacterial abundances (*n* = 144) yielded a mean of ~6 × 10^9^ (log_10_ 9.699 ± 0.255) CFU g/soil across all wheat species (*n* = 19), significantly higher than unplanted soil (*n* = 8) (~3 × 10^9^; log_10_ 9.321 ± 0.403) (two-way ANOVA: *F* = 16.4, df (degrees of freedom) = 1, *P* < .0001) (Fig. 1D). This trend was also seen for the abundance of rhizoplane bacteria with PGP traits with ~2 × 10^9^ (log_10_ 9.084 ± 0.627) CFU g/soil, whereas bacteria with PGP traits from unplanted soil were less abundant (5 × 10^8^; log_10_ 8.075 ± 0.866) (*F* = 24.8, df = 1, *P* < .0001) (Fig. 1E). Fertilization had no impact on total bacterial abundance (*F* = 0.1, df = 1, *P* = .7317) but did on the abundance of rhizoplane bacteria with PGP traits (*F* = 13.3, df = 1, *P* = .0004), which were reduced in fertilized wheat (8.740 ± 0.718) compared with non-fertilized wheat (9.408 ± 0.255) (Tukey, *P* < .0001) (Fig. 1E).

### Combined impact of fertilization and polyploidization on wheat rhizobacterial community structure

**Figure 2:**
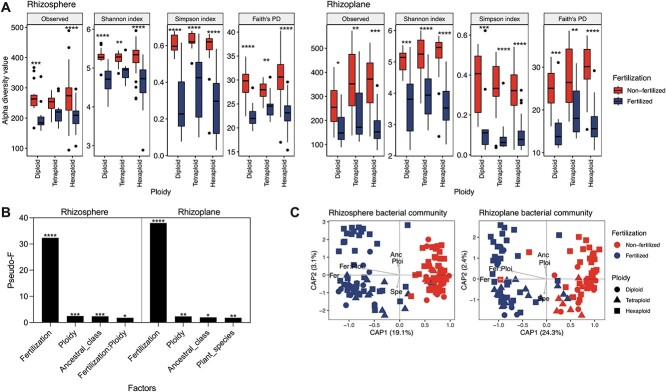
Impact of genome polyploidization and chemical fertilization on rhizobacterial communities; (A) alpha diversity estimates at ASV level, and shown are the median (line) and hinges at first and third quartiles (25th and 75th percentiles); significant differences as determined by two-way ANOVA (type III) tests followed by pairwise comparisons with Tukey correction are shown by “^*^,” “^*^^*^,” “^*^^*^^*^,” and “^*^^*^^*^^*^” for *P* < .05, *P* < .01, *P* < .001, and *P* < .0001, between fertilization groups; (B) pseudo-*F* values for factors influencing rhizobacterial community from PERMANOVA tests (type I, 9999 permutations, non-nested, multifactorial), and all PERMANOVA results can be found in Supplementary Data S2, and the ASV abundance has been standardized by DESeq2 median of ratios; (C) canonical analysis of principal coordinates (CAP) plots; factors (fertilization (Fer), ploidy (Ploi), ancestry class (Anc), and plant species (Spe)) shaping the rhizobacterial community composition and the interaction between them (fertilization and ploidy (Fer:Ploi)) are represented by arrows; the length of the arrow represents the strength each factor has on variation in the microbial community; only factors that significantly contributed to rhizobacterial variation are shown (Supplementary Data S2).

We observed the same trend in response to fertilization across all plant ploidy groups in that application significantly lowered rhizobacterial alpha diversity estimates (two-way ANOVA, *P* < .05) but that no clear differences were seen between ploidy levels (*P* > .05) (Fig. 2A, Data S1, Supplementary Notes: Extended Discussion). Fertilization was also the strongest factor separating bacterial communities in both the rhizosphere and rhizoplane with ploidy, ancestral class, and plant species causing similar weak, yet significant effects (Fig. 2B, Data S2). Furthermore, ploidy and fertilization interactively influenced the rhizobacterial community composition. We employed constrained ordination (CAP), considering only factors that significantly influenced rhizobacterial community abundances based on PERMANOVA (fertilization × ploidy + ancestral class + plant species). Bacterial communities in the rhizosphere and rhizoplane separated primarily due to fertilization followed by ploidy level (Figs 2C and S8).

**Figure 3:**
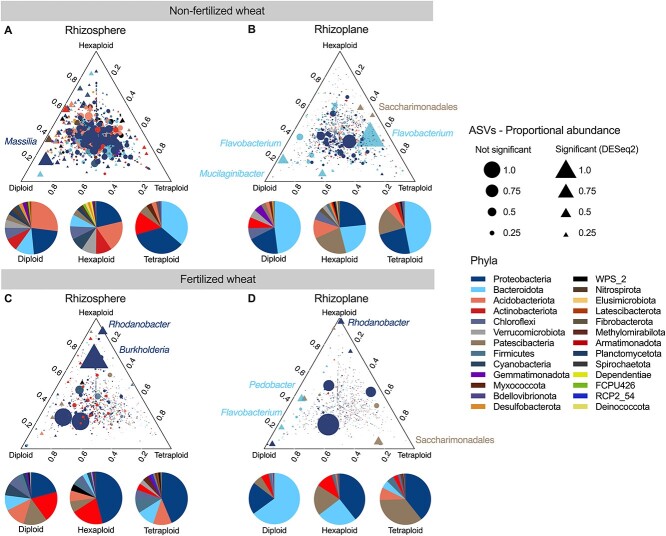
Distribution of rhizobacterial phyla in wheats with different ploidy levels ternary; plots showing relative abundance of all culture-independent bacterial ASVs for diploid, tetraploid, and hexaploid wheats from non-fertilized (A) rhizosphere, (B) rhizoplane, and fertilized (C) rhizosphere, (D) rhizoplane samples, and each point represents an ASV and is colored based on phylum level; the size of each point represents its proportional abundance calculated from normalized counts of the most abundant ASV, and its position represents its relative abundance with respect to each ploidy group, and triangles represent ASVs significantly enriched in one wheat ploidy compared with the others based on DESeq2 differential abundance analysis at a significance level of *P* < .05, adjusted for multiple testing using the Benjamini–Hochberg procedure; circular points represent ASVs that are not significantly enriched in any wheat; pie charts represent the percentage of differentially abundant phyla generated from the cumulative baseMean for ASVs (average of the normalized count values, divided by size factors, taken over all samples); a full list of differentially abundant ASVs can be found in Supplementary Data S4.

Analyzing the microbiota separately for both fertilization and soil compartments showed three distinct rhizobacterial communities for diploid, tetraploid, and hexaploid wheats (PERMANOVA, *P* < .05) (Figs S9 and S10, Data S2). We also found that other factors influenced the bacterial community as well as ploidy level, such as ancestral class, genome, and plant species (PERMANOVA, *P* < .05) (Data S2, Fig. S11). Differential abundance analysis identified ASVs discriminating between ploidy groups (~21% in non-fertilized samples and ~ 29% in fertilized samples) (Wald test, individual *P*-values < .05, Benjamini–Hochberg procedure for multiple testing) (Data S4). In total, 26 phyla were detected within the culture-independent dataset (Fig. 3, Table S3). In non-fertilized rhizoplane samples, the Bacteroidota were enriched in diploid (48%) and tetraploid (47%) compared with hexaploid wheats (23%), whereby key ASVs were classified as *Flavobacterium* and *Mucilaginibacter* (Fig. 3B). Additionally, the Patescibacteria were enriched in the rhizoplane of non-fertilized tetraploid (17%) and hexaploid (22%) compared with diploid (3%) wheats (Fig. 3B). Differences between ploidy groups were notable in fertilized rhizoplane samples; Bacteroidota were abundant in diploid wheats (65% vs. 25% (hexaploid) and 4% (tetraploid)). Comparatively, the Patescibacteria were enriched in tetraploid wheat (35% vs. 19% (hexaploid) and 6% (diploid)), similar to non-fertilized samples (Fig. 3D).

### Fertilization reduces the abundance of rhizoplane bacteria with PGP traits in allopolyploid wheats but not in diploid wild wheat progenitors

**Figure 4:**
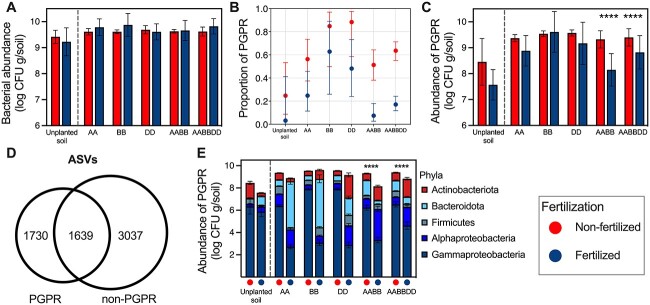
Abundances of rhizoplane bacteria with PGP traits; analysis of culturable bacterial abundances isolated from soil (unplanted and rhizoplane) samples collected from diploid wheat progenitors (AA, BB, DD), tetraploid (AABB), and hexaploid (AABBDD) wheats, grown with and without the addition of NPK fertilizer granules, as well as unplanted control pots; (A) absolute abundance (log CFU g/soil; CFU is colony-forming units) of culturable bacteria isolated from soil samples on 10% TSA; (B) back transformed means with 95% confidence intervals, calculated from negative binomial generalized linear models with genotype and fertilization as factors from the proportion of bacteria with PGP traits, and for full statistical analysis, see Supplementary Data S5; (C) absolute abundance (log CFU g/soil) of bacteria with PGP traits as determined by functional bioassays, and significant differences as determined by two-way ANOVA (type III) post-hoc multiple comparison tests with Šidák correction are shown by “^*^^*^^*^^*^” for *P* < .0001; (D) venn diagram of the culturable bacterial community classified as “PGPR” or “non-PGPR”; numbers indicate the number of shared and unique ASVs; (E) phyla percentages were calculated from 16S rRNA gene ASV counts (PGPR only) which were used to determine the absolute abundance of each phylum based on the abundance of PGPR.

We hypothesized that modern hexaploid wheat varieties, typically grown with fertilizer, have a reduced ability to establish mutualistic root microbiome associations. To test this, we analyzed culturable rhizoplane bacteria based on fertilizer application and plant genotype: wild wheat progenitors (AA, BB, DD) and allopolyploid wheats (AABB, AABBDD). Differences in the absolute abundance of culturable rhizoplane bacteria isolated from these wheats were not influenced by fertilization or genotype (two-way ANOVA, *P* > .05) (Fig. 4A). We functionally tested a large subset of these culturable isolates (~15 000) to identify putative PGPR abundances based on nutrient solubilization traits (Fig. S12, Supplementary Notes: Result S2), determining 39% of isolates to exhibit PGP traits. Here, any isolate which tested positive for any two of the five traits tested (casein hydrolyzation, phosphate, potassium, and zinc solubilization, and siderophore production) was defined as a putative PGPR (Fig. S2). The proportion of putative PGPR was influenced by both fertilization (analysis of deviance, df = 1, deviance = 31.5, residual df = 145, residual deviance = 44.7, *P* < .0001) and genotype (df = 5, deviance = 9.5, residual df = 146, residual deviance = 76.3, *P* < .0001). Proportional PGPR abundances were higher in non-fertilized wheats, with *Aegilops* species showing the highest proportions both with and without fertilizer treatment compared with the lowest proportions of PGPR which were isolated from fertilized unplanted soil, AABB and AABBDD wheats (Fig. 4B). Full statistical analysis and results are detailed in Data S5. Additionally, the absolute abundance of rhizoplane bacteria with PGP traits was statistically influenced by fertilization (two-way ANOVA: *F* = 14.6, df = 1, *P* = .0002), genotype (*F* = 5.3, df = 4, *P* = .0005), and their interaction (*F* = 2.9, df = 4, *P* = .0237), with post-hoc tests revealing that this variation was caused by allopolyploid wheats (AABB, AABBDD) (Šidák, *P* < .0001), whereas there were no statistical differences between absolute abundances of rhizoplane bacteria with PGP traits from non-fertilized vs. fertilized diploid wild wheat progenitors (AA, BB, DD) (*P* > .05) (Fig. 4C). We classified the taxonomic composition of rhizoplane bacteria with PGP traits compared with rhizoplane bacteria with no PGP traits. We acknowledge that our functional tests were not comprehensive for all PGP traits but in the context of this study we will be referring to isolates which tested positive for functional traits as “PGPR” and vice versa as “non-PGPR.” Amplicon sequencing of 16S rRNA genes (Fig. S3) yielded ~6400 ASVs, 27% PGPR, 47% non-PGPR (Fig. 4D). Proportional abundances of phyla from: (i) all isolates and (ii) isolates with PGP traits were used to determine the absolute abundance of phyla for (i) total bacterial abundance (Fig. S13) and (ii) putative PGPR abundance (Fig. 4E). In general, for the isolates with PGP traits, Gammaproteobacteria were enriched in all non-fertilized samples (two-way ANOVA: *F* = 2015, df = 1, *P* < .0001), except for in the unplanted soil control, where there were no differences (Šidák, *P* > .05) (Fig. 4E). Bacteroidota were enriched in wild wheat progenitors (AA, BB, DD) treated with fertilizer in comparison to allopolyploid wheats (Tukey, *P* < .0001) (Fig. 4E).

### Diploid wheats select for Bacteroidota with PGP traits despite fertilization

**Figure 5:**
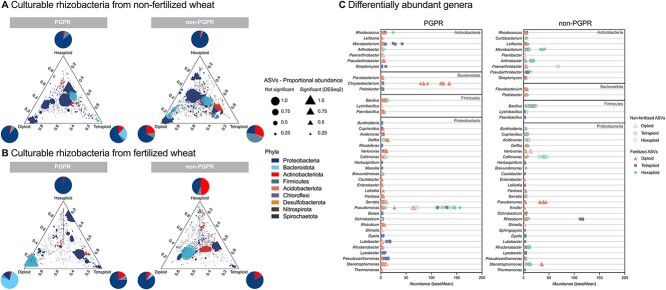
Selection of rhizoplane bacteria with PGP traits; ternary plots showing relative abundance of all bacterial ASVs for diploid, tetraploid, and hexaploid varieties for “PGPR” and “non-PGPR” isolated from (A) non-fertilized and (B) fertilized rhizoplane samples; each point represents an ASV and is colored based on phylum level, and the size of each point represents its proportional abundance calculated from normalized counts of the most abundant ASV; its position represents its relative abundance with respect to each ploidy group; triangles represent ASVs significantly enriched in one wheat ploidy compared with the others based on DESeq2 differential abundance analysis at a significance level of *P* < .05, adjusted for multiple testing using the Benjamini–Hochberg procedure; circular points represent ASVs that are not significantly enriched in any wheat; pie charts represent the percentage of differentially abundant phyla generated from the cumulative baseMean for ASVs (average of the normalized count values, divided by size factors, taken over all samples), and (C) differentially abundant genera enriched in wheats are displayed based on their abundance (baseMean) from DESeq2 analysis, and points represent individual ASVs, ordered based on phylum level, color coded based on ploidy level for non-fertilized (outlines) and fertilized (filled) points; a full list of differentially abundant ASVs can be found in Supplementary Data S6.

**Figure 6:**
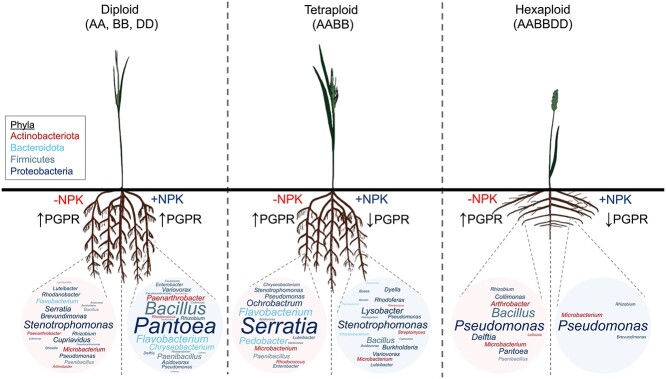
PGPR selection has reduced with agricultural intensification; in this schematic representation, the number of enriched genera with PGP traits (word clouds) associated with wheats dramatically reduced with genome polyploidization and fertilization (+NPK), and word clouds were made using wordart.com from differentially abundant ASVs classified to genus level associated with each ploidy level and fertilization type (Supplementary Data S6); genera are color coded by phyla; the changes in structure of wheat root systems due to domestication were not the focus of this study but were based on previous literature [18, 78, 79].

Continuing the analysis of taxonomic shifts in PGPR abundances caused by polyploidization, differential abundance analysis was performed to identify PGPR ASVs highly associated with diploid, tetraploid, and hexaploid wheats grown with and without fertilizer. Analysis revealed 20% (263 ASVs), 11% (144 ASVs), and 5% (70 ASVs) of PGPR to be enriched in the rhizoplane of diploid, tetraploid, and hexaploid wheats, respectively (Data S6). Differentially abundant PGPR phyla were mostly dominated by Proteobacteria (between 59% and 97%) except for fertilized diploid wheats where the Bacteroidota dominated (77%) (Fig. 5A and B, PGPR), mainly represented by *Chryseobacterium* (Fig. 5C, PGPR). Taxonomic composition of differentially abundant ASVs varied between non-PGPR and PGPR isolates in a fertilization-dependent manner (Fig. 5). For example, in fertilized diploid wheat, Bacteroidota abundance decreased by 70% (Fig. 5B, non-PGPR). These findings correlate with the culture-independent differential abundance analysis but with some key differences. Firstly, only 3.5% of phyla detected in the culture-independent dataset were represented by the culture-dependent dataset (26 vs. 9 phyla, respectively), emphasizing the taxonomic gap between culturable and readily culturable bacteria. A key point considering this would be that there were no culturable isolates identified as Patescibacteria (Fig. 5A and B); the highly abundant phyla present in tetraploid and hexaploid wheat rhizoplanes (Fig. 3B and C). The rhizoplane of fertilized diploid wheats however showed similar taxonomic profiles in both datasets. In the culture-independent analysis, Bacteroidota was the most abundant phylum (65%) (Fig. 3D), reflecting findings in culture-dependent analysis (Fig. 5B). The culture-dependent dataset highlighted *Chryseobacterium* as the prevalent genus in this group (Fig. 5C), whereas the culture-independent dataset indicated *Flavobacterium* and *Pedobacter* as abundant genera (Fig. 3D), though all three genera were present in both datasets (Data S4 and S6). Additionally, the complexity of the supported PGPR populations was reduced in polyploid wheat (Fig. 6). For example, there were half the number of genera differentially more abundant in hexaploid wheats (10 genera) compared with diploid wheats (20 genera) (Fig. 6). Furthermore, hexaploid wheats grown under fertilization had a higher number of *Pseudomonas* ASVs compared with other ploidy levels but low abundances of only three other genera (*Rhodococcus, Brevidimonas*, and *Rhizobium*), whereas diploid wheat selected for 21 genera with a high number of *Pantoea*, *Bacillus*, and *Flavobacterium* (Fig. 6).

## Discussion

We explored whether aboveground selection for high yields during wheat domestication and modern breeding had any unintended impact on the selection and assembly of rhizobacterial communities. We approached this by performing a pot experiment in soil under contrasting fertilization conditions. Our aim was to establish the effect that chemical fertilizer introduction during the Green Revolution had on plant-microbe interactions in wheat by comparing the rhizobacterial community of wild wheat progenitors through to modern varieties. This allowed us to characterize the influence of modern bread wheat lines, their wild ancestors, and domesticated species, on the structure of the rhizobacterial communities. The importance of root-associated microorganisms in supporting plant growth under suboptimal growth conditions has been well documented [57–59]. Thus, the potential differential ability of plant accessions to select beneficial microorganisms to the root environment when grown in a nutrient poor soil, relative to nutrient replete conditions, over the course of domestication and breeding, was investigated.

As expected, fertilization was the dominant factor influencing rhizobacterial diversity, community, and taxonomic composition, and abundances of culturable rhizoplane bacteria with PGP traits (Fig. 1). These results reaffirm that chemical fertilization significantly alters the root-associated microbiota [60–65], including reducing rhizobacterial diversity and selection of PGPR [37], but here, we demonstrate this across a wide range of wheat species (Fig. 2A). Even though this same trend has been demonstrated in bulk soil [66–70], our results show bacterial community alpha diversity to be unaffected by fertilization in unplanted soil pots (Fig. 1A), likely due to the low carbon levels in our bare fallow soil system (Table S2) and the experimental setup (pot experiment over one growth season). For the purpose of this study, the similarity of rhizobacterial communities in unplanted soil with and without fertilizer magnifies the effect that wheat plants have over the assembly and selection of the rhizo-microbiome and contributes to the suitability of this system for studying plant–microbe interactions. Additionally, there was a significantly lower abundance of rhizoplane bacteria with PGP traits isolated from unplanted soil and no difference in abundance under contrasting fertilization conditions (Fig. 1E). This further confirms the experimental method as suitable for assessing the abundance of putative PGPR in the root environment as well as the effect of fertilization on plant selection of PGPR. Despite fertilized wheats having a much higher plant aerial biomass than non-fertilized wheats (Fig. S7A), the rhizobacterial community diversity was significantly lower (Fig. 1A), which is not necessarily attributed to a detrimental microbiome, but here, we also found a reduced abundance of culturable rhizoplane bacteria with PGP traits in fertilized wheats (Fig. 1E), whereas the overall abundance of rhizobacteria was not affected by fertilization (Fig. 1D).

In comparison, we observed three distinct rhizobacterial communities for diploid, tetraploid, and hexaploid wheats in the rhizosphere and rhizoplane of non-fertilized and fertilized wheats (Figs S9 and S10). Similar trends have previously been shown for prokaryotic community composition of genetic groups of modern cultivars and ancestral landraces for wheat [25], maize [71], and rice [72]. We also found that other factors influenced the bacterial community as well as ploidy level, such as ancestral class and plant species (Figs 2B and S11), in agreement with previously published observations [22]. Furthermore, we found that ploidy level and fertilization interactively influenced the rhizobacterial community composition (Fig. 2C, Data S2) suggesting that microbial communities in the root environment are shaped by interactions between agricultural management practices such as fertilization conditions, and host selection processes which are dependent on genomic content [28, 73].

PGPR provide their host with enhanced access to mineral nutrients [74], representing an attractive alternative to soil-degrading chemical fertilizers [75]. PGPR selection by the host is the result of a step-wise selective process, the composition and function of which are controlled, in part, by the plant genome [76]. The human footprint on the evolutionary history of cultivated plants can be evidenced through phenotypical changes from manmade selection processes that differentiated modern varieties from their wild relatives [16]. This also appears to have impacted plant-associated microorganisms [77], notably by the preferential association of members of the Actinobacteriota phylum with the modern varieties opposed to the preferential association of members of the Bacteroidota phylum with wild relatives [78, 79]. In addition, interventions operated by farmers also impact plant–microbiota interactions [80, 81]. In this study, we show how fertilization changes rhizobacterial selection in diploid wheat progenitors compared with polyploid wheats. We found that: taxonomic composition differed based on wheat ploidy level (Fig. 3 and 5); proportional and absolute abundances of rhizobacteria with PGP traits were reduced in polyploid wheats grown under fertilization (Fig. 4B and 4C); the complexity of the PGPR populations, i.e. number of genera, selected in both the presence and absence of fertilization was reduced particularly in hexaploid wheats (Fig. 6).

The depletion of rhizoplane bacteria with PGP traits in modern accessions (Fig. 6) suggests that the Green Revolution, which relied on chemical fertilizers, has caused a reduced association between hexaploid wheat and their beneficial rhizosphere microbiota. Considering that modern crops have been selected to respond to high inputs of synthetic fertilizers, rather than the establishment of beneficial plant–microbiota associations, it is legitimate to hypothesize that reduced plant genotypic selection of PGPR in polyploid wheat under high fertilization conditions is a consequence of the selection for yield or other macroscopic traits. Furthermore, it has been shown that fertilization markedly affects gene expression in wheat [82]. We propose that fertilization depletion differentially influences plant gene expression in diploid compared with polyploid accessions. It follows that differential wheat gene expression, possibly linked to root exudate production, could play a role in the reduced selection of rhizobacteria with PGP traits in fertilized tetraploid and hexaploid wheats, which would further support the hypothesis that hexaploid wheat is more dependent on fertilization in comparison to ancestral landraces where interactions with soil microbes are crucial for holobiont fitness [83].

Important PGP traits that were not included in this study include those related to disease suppression, enzymatic activity, and abiotic stress tolerance. However, other studies have revealed interesting impacts that domestication has also had on these traits. For example, in terms of disease suppression, it has been shown that wheat domestication likely caused a reduction of the natural biocontrol potential of rhizosphere-associated bacteria against pathogenic fungi [30], also demonstrated by the reduction of pathogenic fungal taxa in the rhizosphere of wild compared with modern wheats [21]. Related to nutrient cycling, further to our observations, it was previously shown that domestication shifted the rhizosphere microbiome in wild tetraploid wheats from a community enriched for carbon fixation, nitrogen transformation, and phosphorous mineralization to one enriched in carbon degradation, inorganic nitrogen fixation, and inorganic phosphorous solubilization in domesticated tetraploid wheats, and that this was controlled by different root exudates [29]. Additionally, a recent study demonstrated higher urease activity in the rhizosphere of wild wheat species compared with domesticated wheat species and that alkaline phosphomonoesterase activity was selectively higher in wild wheat species dependent on field site location [30]. Our results show that similar absolute abundances of rhizobacteria with PGP traits were isolated from all wheats under non-fertilized conditions, and that the addition of chemical fertilizer caused a reduction of PGPs in polyploid wheats (Figs 4C). This was combined with a gradual loss of genera represented by these isolated rhizobacteria from diploid to hexaploid wheats (Fig. 6). Therefore, we propose that changes in the wheat genome brought on by polyploidization events (pleiotropy, linkage, genetic drift) as well as changes brought about by agricultural intensification in terms of management practices have contributed to these changes.

The main result of the present study is that diploid wild wheats selected for Bacteroidota which was observed in both culture-independent and -dependent datasets (Figs 3–6). As mentioned previously, this is not the first case that the Bacteroidota have been associated with wild plant species [78]. Bacteroidota represent an important group of soil bacteria that possess many beneficial functions such as suppression of plant diseases [84] and may have important implications for future crop breeding to reduce pathogen susceptibility [85]. Additionally, Bacteroidota are key contributors to soil nutrient cycles, particularly organic phosphorous [84]. In fact, plant-associated Bacteroidota (e.g. *Flavobacterium* spp.) have been shown to have superior phosphatase activity when compared with other rhizobacteria, such as *Pseudomonas* spp., in both the presence and absence of inorganic phosphate [86]. This phenotype is due to the presence of a unique phosphate-insensitive phosphatase PafA in the Bacteroidota which is not silenced by high concentrations of available P [87]. In this study, we show that wild wheats select for Bacteroidota with nutrient acquisition traits such as phosphate solubilization under both fertilizer-depleted and -replete conditions (Figs S4E and 5B) with genera identified as *Flavobacterium* and *Chryseobacterium* (Fig. 5C)*.* The unique organophosphorus utilization machinery that the Bacteroidota possess provides a mechanism that explains the higher abundances of Bacteroidota in fertilized wild wheat, enabling species to grow on organophosphorus compounds as a sole carbon and energy source when high exogenous levels of P render classical phosphatases inactive. This compared with tetraploid and hexaploid wheats which selected for high abundances of Proteobacteria (Fig. 5B), particularly *Pseudomonas* spp. under fertilization (Fig. 5C), which could be linked to the greater production of simple sugar root exudates in modern wheat varieties compared with ancient wheat cultivars [88], which may contribute to a competitive advantage and a concomitant higher abundance of Proteobacteria in these cultivars. In fact, metagenomic analyses revealed an enrichment of *Pseudomonas* and an increase in functional genes important for *Pseudomonas* accumulation in the rhizosphere of tetraploid domesticated wheat compared with wild tetraploid wheat [29]. Additionally, this study [29], which grew wheat varieties under fertilization conditions, showed an increase in the Bacteroidota genus *Pedobacter* in wild tetraploid wheats which we also found in fertilized tetraploid wheat along with an enrichment of *Pseudomonas* (Fig. 5C). The presence of *pafA* in the Bacteroidota could provide an explanation as to why there were no differences in the abundance of the alkaline phosphatase encoding *phoX* gene in the rhizosphere of wild and domesticated wheats [30]; it would be interesting to measure the abundance of *pafA* in this scenario to test whether abundances are higher in wild wheats where the Bacteroidota are likely to be more abundant. Xyloglucan, a high molecular weight root exudate, is secreted by a range of plants including wheat but was potentially important in the initial colonization of land plants due to its role in soil structure and functioning as a soil aggregator [89]. Given that the Bacteroidota have the ability to degrade complex biopolymers, soluble xyloglucans and high molecular weight exudates may be important sources of nutrients for plant-associated *Flavobacterium* spp. when invading the rhizosphere, particularly during early land colonization. This could suggest that the Bacteroidota are key early microbial colonizers of plants as indicated by their high abundances in the rhizosphere of wild sugar beet [90], barley [91], lettuce [92], rice [93], common bean grown in agricultural soil [94], other wild plant species [78], and now wheat (supported by [26, 28]). This combination of complex biopolymer utilization and organophosphorus utilization provides a distinct metabolic niche for Bacteroidota and facilitates the coexistence with other rhizobacteria that specialize in the acquisition of low molecular weight liable C such as *Pseudomonas* and Burkholderiales. Furthermore, the high importance of Bacteroidota in nutrient acquisition is not limited to plants. *Bacteroides* are key members associated with gut health in humans due to their evident nutrient acquisition capacity [95], thus cementing the notion that this phylum has cross-Kingdom significance in terms of promoting eukaryotic host health [96].

## Conclusions

Understanding the functional importance of microbes that may have been lost due to domestication is instrumental to plant breeding programs and for improving future crop production systems [97], and here we identify some of these “missing” microbes to be members of the Bacteroidota, *Flavobacterium* and *Chryseobacterium*, with PGP, nutrient solubilizing traits. Microbiota of crop ancestors may offer a way to enhance sustainable food production, as discussed by the rewilding hypothesis [98]. Future work will involve performing what is known as a microbiome transplant whereby the microbes we have identified as being lost through domestication will be inoculated to modern wheat varieties to identify whether beneficial effects from these microbes can be re-established, which has already been demonstrated [99], analogous to fecal microbiota transplantation to redirect the dysbiotic composition of the human microbiome. Alternatively, the reintroduction of key genetic elements to modern plants from their ancestors, by selective breeding or gene editing, for the selection of beneficial microbiota from the bulk soil reservoir is another promising strategy. These approaches could result in improved rhizosphere-beneficial microbe associations in modern wheat, less dependent on fertilizer application. It would also be interesting to test whether similar results are obtained under organic fertilizer amendments. Taken together, this study advances the current understanding of crop–microbiota interactions in terms of host genotype and environmental drivers among an evolutionary assemblage of wheat species grown with and without chemical fertilizer, as well as identifying microbes with PGP traits that may have been lost as a result of agricultural intensification. Understanding these changes has the potential to open new avenues to identify and promote beneficial interactions, toward ecologically intensified agroecosystems and more sustainable, lower input agriculture whereby plant and soil are given equal status.

## Supplementary Material

Supporting_Information_wrae131

Supplementary_Notes_wrae131

Supplementary_Data_wrae131

## Data Availability

Raw sequences can be accessed in the Sequence Read Archive of NCBI under accession no. PRJNA1013055. All processed datasets have been deposited under the CC-BY 4.0 license in a Rothamsted Repository (DOI https://doi.org/10.23637/rothamsted.98zz7). R scripts for the full analyses are freely available under the Apache 2–0 license and have been deposited in a Zenodo repository (DOI https://doi.org/10.5281/zenodo.12731905).
