# Supplementary material for: Agricultural intensification reduces selection of putative plant growth-promoting rhizobacteria in wheat
Source: ISME J. 2024 Jul 11;18(1):wrae131. doi: 10.1093/ismejo/wrae131 (PMC11292143; doi:10.1093/ismejo/wrae131)
Supplement: Supporting_Information_wrae131 [file supporting_information_wrae131.pdf]

**Supporting Information**

# Agricultural intensification reduces selection of putative plant growth-promoting rhizobacteria in wheat

Tessa E. Reid[1,2], Vanessa N. Kavamura[1], Adriana Torres-Ballesteros[1], Monique E. Smith[1,3], Maïder Abadie[1,4+], Mark Pawlett[2], Ian M. Clark[1], Jim A. Harris[2] and Tim H. Mauchline[1*]

[1]Sustainable Soils and Crops, Rothamsted Research, Harpenden, Hertfordshire, AL5 2JQ, UK
[2]School of Water, Energy and Environment, Cranfield University, Cranfield, Bedfordshire MK43 0AL, UK
[3]Department of Ecology, Swedish University of Agricultural Sciences, Uppsala, SE-750 07, Sweden
[4]INRAE, UR1264 MycSA, CS2032, 33882 Villenave d'Ornon, France

+Current affiliation

*Corresponding author: Dr Tim H. Mauchline, Rothamsted Research, Harpenden, AL5 2JQ

tim.mauchline@rothamsted.ac.uk

**Contents**

**1. Figures**

**Fig. S1.** The evolution of plant breeding in wheat.

**Fig S2**. Functional bioassay analysis to identify bacterial isolates with plant growth-promoting (PGP) traits.

**Fig. S3.** PGPR (plant growth-promoting rhizobacteria) vs. non-PGPR amplicon sequencing.

**Fig. S4.** Two-factor randomized block design of experimental plant layout with fertilization (UnTreated and Treated) and plant variety (*A. speltoides, A. tauschii,* Bulk soil, *T. aestivum* (cv.: Avalon, Cadenza, Chidham, Crusoe, Gallant, Hereward, Malacca, Red Lammas, Victor), *T. carthlicum, T. dicoccoides, T. macha, T. monococcum, T. polonicum, T. spelta, T. turanicum, T. urartu)* as factors.

**Fig. S5.** Photos of wheat species used in this study, taken at flowering stage.

**Fig. S6.** Rarefaction curve analyses of bacterial species richness as a function of sequencing depth for (A) all samples and (B) samples at a cut-off of 2,000 reads as used for downstream alpha diversity analysis for culture-independent samples.

**Fig. S7.** Average dry plant biomass (A), ear length (B), and height (C) of wild wheat progenitors (AA, BB, DD) and allopolyploid (AABB, AABBDD) wheats grown in nutrient-depleted agricultural soil with and without fertilizer addition.

**Fig. S8.** Principal Coordinate Analysis (PCoA) plots of bacterial community based on Bray-Curtis distance in non-fertilized and fertilized rhizosphere (A) and rhizoplane (B) samples from diploid, tetraploid and hexaploid wheat varieties.

**Fig. S9.** Canonical Analysis of Principal coordinates (CAP) plots of bacterial composition based on Bray-Curtis distance with ploidy level as the factor of constraint; *p*-values are from permutation tests (ANOVA; capscale under a reduced model).

**Fig. S10.** PCoA plots of bacterial community based on Bray-Curtis distance in non-fertilized (A) and fertilized (B) rhizosphere and rhizoplane samples from diploid, tetraploid and hexaploid wheat varieties.

**Fig. S11.** CAP analysis of the bacterial rhizosphere (A, E, I) and rhizoplane (B, F, J) community from non-fertilized wheat (first two columns) and the bacterial rhizosphere (C, G, K) and rhizoplane (D, H, L) community for fertilized wheat (last two columns) using ancestral

class (A-D), genome (E-H), and plant species (I-L) as the factors of constraint; *p*-values are from permutation tests (ANOVA; capscale under a reduced model).

**Fig. S12.** Analysis of culturable bacterial abundances isolated from soil (unplanted and rhizoplane) samples collected from diploid wheat progenitors (AA, BB, DD), tetraploid (AABB) and hexaploid (AABBDD) wheats, grown with and without the addition of NPK fertilizer granules, as well as unplanted control pots.

**Fig. S13.** Classification of phyla abundances in culturable bacterial communities isolated from soil (unplanted and rhizoplane) from diploid wheat progenitors (AA, BB, DD), tetraploid (AABB) and hexaploid (AABBDD) wheats, grown with and without the addition of NPK fertilizer granules, as well as unplanted control pots.

## 2. Tables

**Table S1.** Wheat species chosen for the current study.

**Table S2.** Soil properties from Woburn bare fallow soil sampled in April 2019.

**Table S3.** Phyla from culture-independent amplicon sequence variant (ASV) datasets from non-fertilized rhizosphere, rhizoplane, and fertilized rhizosphere, rhizoplane wheat samples.

## 3. Data

**Data S1.** Statistical analysis of alpha diversity metrics.
See the **Supplementary Data file**: Data S1 (XLSX).

**Data S2.** PERMANOVA results.
See the **Supplementary Data file**: Data S2 (XLSX).

**Data S3.** Data used for Figure 1C. Culture-independent 16S rRNA gene ASVs that were tested by DeSeq2 to determine ASVs enriched in non-fertilized vs fertilized wheats from unplanted, rhizosphere, and rhizoplane soil samples.
See the **Supplementary Data file**: Data S3 (XLSX).

**Data S4.** Data used for Figure 3A-D. Culture-independent 16S rRNA gene ASVs that were tested by DeSeq2 to determine ASVs enriched in diploid, tetraploid, and hexaploid wheats grown in soils with or without the addition of fertilizer. Tests were performed separately for each group: (1) non-fertilized rhizosphere, (2) non-fertilized rhizoplane, (3) fertilized rhizosphere, (4) fertilized rhizoplane.
See the **Supplementary Data file**: Data S4 (XLSX).

**Data S5.** Statistical analysis of proportional abundances of isolates positive for each functional bioassay.
See the **Supplementary Data file**: Data S5 (XLSX).

**Data S6.** Data used for Figure 5 and 6. Culture-dependent 16S rRNA gene ASVs that were tested by DeSeq2 to determine ASVs enriched in diploid, tetraploid, and hexaploid wheats grown in soils with or without the addition of fertilizer. Tests were performed separately for each group: (1) non-fertilized PGPR, (2) non-fertilized non-PGPR, (3) fertilized PGPR, (4) fertilized non-PGPR.
See the **Supplementary Data file**: Data S6 (XLSX).

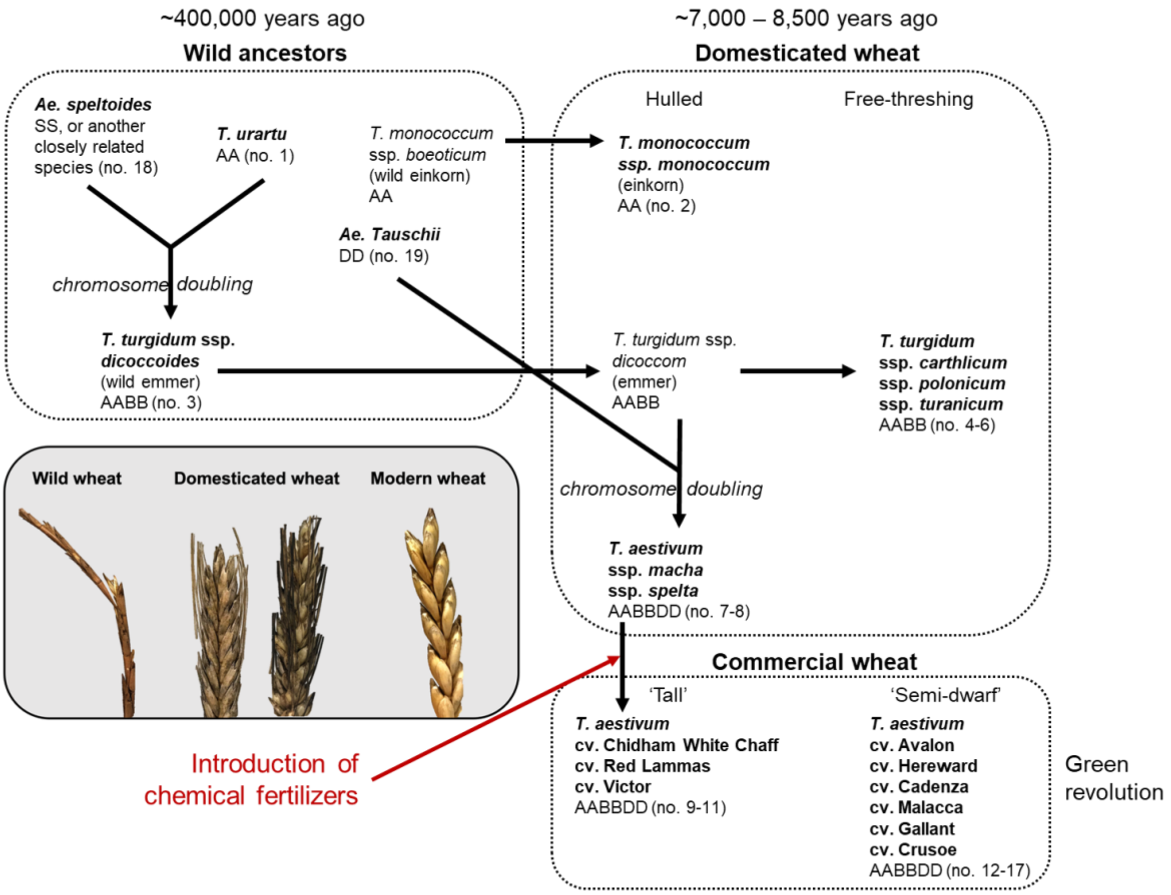


**Fig. S1.** The evolution of plant breeding in wheat. Wild tetraploid wheat, *Triticum turgidum* ssp. *dicoccoides* originated approximately 400,000 years ago from the polyploidization of two closely related diploid species, most likely *Aegilops speltoides* [1-5] and *T. urartu* [6] contributing the A and B portions of the AABB genome. Wild relatives of wheat were cultivated over a thousand-year period resulting in domesticated diploid *T. monococcum* (Einkorn), and tetraploid *T. turgidum* ssp. *dicoccom* (Emmer), *carthlicum*, *polonicum*, and *turanicum*. The final hybridization event between tetraploid wheat (AABB) and *Ae. tauschii* (DD) is thought to have occurred only once or twice, ~8000–10,000 years ago, resulting in the hexaploid (AABBDD) wheat species, *T. aestivum* [5], most used in the production of bread including ssp. *macha* and *spelta*. Significant yield gains were achieved by wheat breeders via the exploitation of genetic variation that arose via gene mutation resulting in commercial cultivars *T. aestivum* cv. Chidham White Chaff (1790), Red Lammas (1850), and Victor (1908). During the Green revolution (1960s), mutant alleles of the *Reduced height* (*Rht*) dwarfing genes [7] were introduced into modern wheat cultivars resulting in plant height reduction which led to the commercial cultivars Avalon (1980), Hereward (1989), Cadenza (1992), Malacca (1997), Gallant (2009), and Crusoe (2012). Text highlighted in bold represent cultivars used in the study (adapted from Tkacz et al. [8]); numbers refer to Table S1.

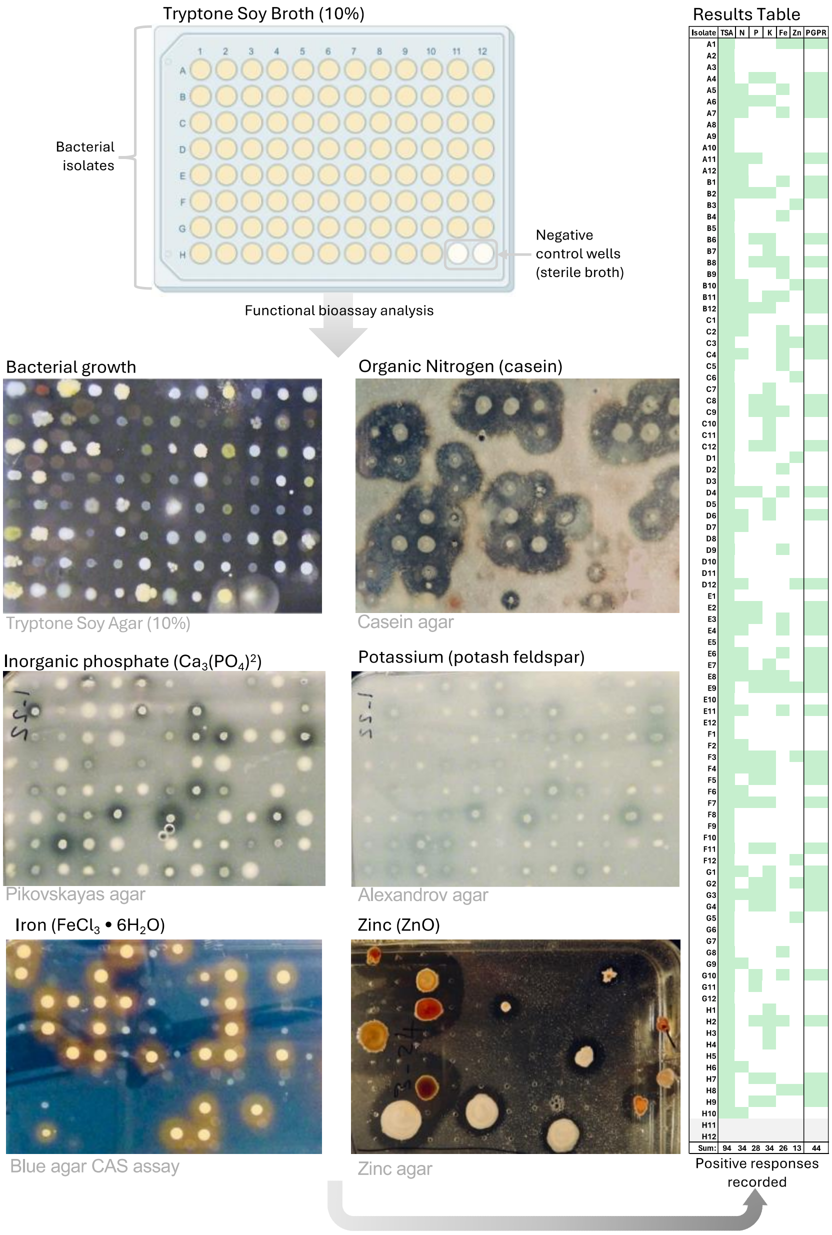


**Fig S2.** Functional bioassay analysis to identify bacterial isolates with plant growth-promoting (PGP) traits. Diagram depicts methodology for one soil sample. Colonies previously picked from diluted rhizoplane samples spread on agar were grown in 10% tryptone soya broth in 96-well plates; 94 colonies were picked with one colony per well, wells H11 and H12 were left uninoculated as negative control wells. Isolates were then spot inoculated using a 96-prong inoculating manifold onto: 10% tryptone soya agar for confirmation of bacterial growth, casein agar [9], Pikovskaya agar [10], Aleksandrov agar with potash feldspar as the potassium source [11], chrome azurol S (CAS) agar [12, 13], and zinc agar (HiMedia M2023) to test for casein, phosphate, potassium, iron, and zinc solubilization, respectively. Positive responses were recorded in the depicted table format and isolates testing positive for any two of the five traits tested was defined as a putative PGPR. The 96-well plate image is from biorender.com.

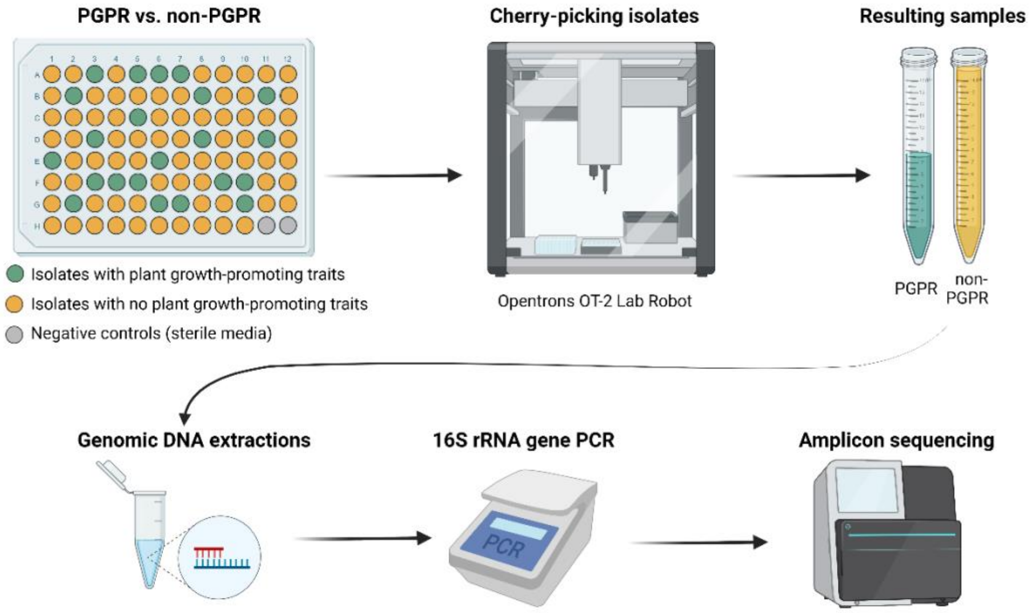


**Fig. S3.** PGPR (plant growth-promoting rhizobacteria) vs. non-PGPR amplicon sequencing. Isolates previously identified to exhibit plant growth-promoting traits (PGPR; depicted in green) vs. isolates that displayed no functional traits (non-PGPR; depicted in yellow) were pipetted (100 µl culture) from wells in a 96-well plate using custom scripts created on the Opentrons protocol designer (https://designer.opentrons.com) on the Opentrons OT-2 Lab Robot (Opentrons, Long Island City, NY, USA) and combined into single tubes, one for PGPR isolates and one for non-PGPR isolates. These were then subjected to genomic DNA extraction (for Gram-positive bacteria) and amplicon sequencing. Figure was created in biorender.com.

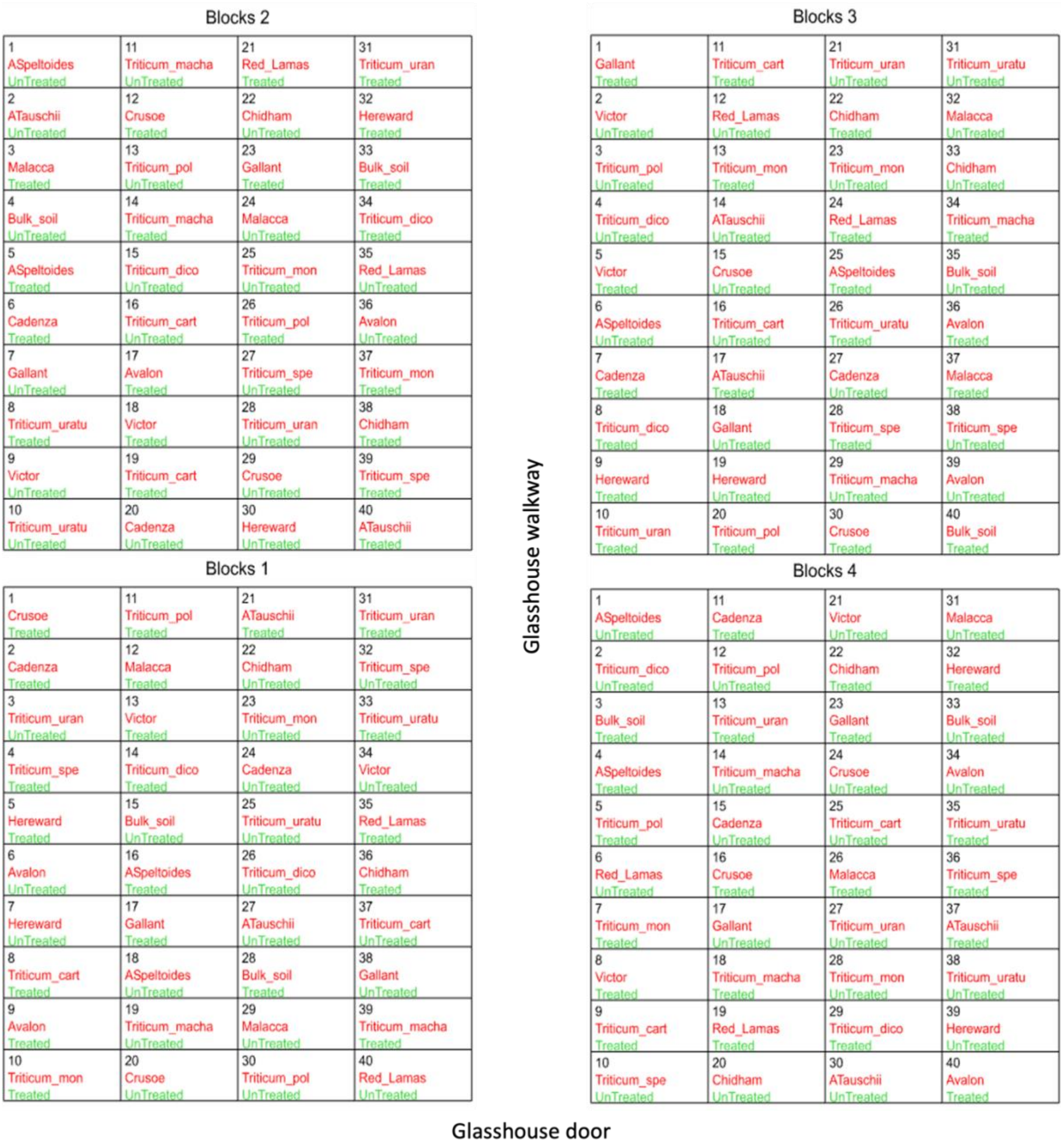

Blocks 2

| | | | |
|---|---|---|---|
| 1 ASpeltoides UnTreated | 11 Triticum_macha UnTreated | 21 Red_Lamas Treated | 31 Triticum_uran Treated |
| 2 ATauschii UnTreated | 12 Crusoe Treated | 22 Chidham UnTreated | 32 Hereward Treated |
| 3 Malacca Treated | 13 Triticum_pol UnTreated | 23 Gallant Treated | 33 Bulk_soil Treated |
| 4 Bulk_soil UnTreated | 14 Triticum_macha Treated | 24 Malacca UnTreated | 34 Triticum_dico Treated |
| 5 ASpeltoides Treated | 15 Triticum_dico UnTreated | 25 Triticum_mon UnTreated | 35 Red_Lamas UnTreated |
| 6 Cadenza Treated | 16 Triticum_cart UnTreated | 26 Triticum_pol Treated | 36 Avalon UnTreated |
| 7 Gallant UnTreated | 17 Avalon Treated | 27 Triticum_spe UnTreated | 37 Triticum_mon Treated |
| 8 Triticum_uratu Treated | 18 Victor Treated | 28 Triticum_uran UnTreated | 38 Chidham Treated |
| 9 Victor UnTreated | 19 Triticum_cart Treated | 29 Crusoe UnTreated | 39 Triticum_spe Treated |
| 10 Triticum_uratu UnTreated | 20 Cadenza UnTreated | 30 Hereward UnTreated | 40 ATauschii Treated |

Blocks 3

| | | | |
|---|---|---|---|
| 1 Gallant Treated | 11 Triticum_cart Treated | 21 Triticum_uran UnTreated | 31 Triticum_uratu UnTreated |
| 2 Victor UnTreated | 12 Red_Lamas UnTreated | 22 Chidham Treated | 32 Malacca UnTreated |
| 3 Triticum_pol UnTreated | 13 Triticum_mon Treated | 23 Triticum_mon UnTreated | 33 Chidham UnTreated |
| 4 Triticum_dico UnTreated | 14 ATauschii UnTreated | 24 Red_Lamas Treated | 34 Triticum_macha Treated |
| 5 Victor Treated | 15 Crusoe UnTreated | 25 ASpeltoides Treated | 35 Bulk_soil UnTreated |
| 6 ASpeltoides UnTreated | 16 Triticum_cart UnTreated | 26 Triticum_uratu Treated | 36 Avalon Treated |
| 7 Cadenza Treated | 17 ATauschii Treated | 27 Cadenza UnTreated | 37 Malacca Treated |
| 8 Triticum_dico Treated | 18 Gallant UnTreated | 28 Triticum_spe Treated | 38 Triticum_spe UnTreated |
| 9 Hereward Treated | 19 Hereward UnTreated | 29 Triticum_macha UnTreated | 39 Avalon UnTreated |
| 10 Triticum_uran Treated | 20 Triticum_pol Treated | 30 Crusoe Treated | 40 Bulk_soil Treated |

Glasshouse walkway

Blocks 1

| | | | |
|---|---|---|---|
| 1 Crusoe Treated | 11 Triticum_pol Treated | 21 ATauschii Treated | 31 Triticum_uran Treated |
| 2 Cadenza Treated | 12 Malacca Treated | 22 Chidham UnTreated | 32 Triticum_spe UnTreated |
| 3 Triticum_uran UnTreated | 13 Victor Treated | 23 Triticum_mon UnTreated | 33 Triticum_uratu Treated |
| 4 Triticum_spe Treated | 14 Triticum_dico Treated | 24 Cadenza UnTreated | 34 Victor UnTreated |
| 5 Hereward Treated | 15 Bulk_soil UnTreated | 25 Triticum_uratu UnTreated | 35 Red_Lamas Treated |
| 6 Avalon UnTreated | 16 ASpeltoides Treated | 26 Triticum_dico UnTreated | 36 Chidham Treated |
| 7 Hereward UnTreated | 17 Gallant Treated | 27 ATauschii UnTreated | 37 Triticum_cart UnTreated |
| 8 Triticum_cart Treated | 18 ASpeltoides UnTreated | 28 Bulk_soil Treated | 38 Gallant UnTreated |
| 9 Avalon Treated | 19 Triticum_macha UnTreated | 29 Malacca UnTreated | 39 Triticum_macha Treated |
| 10 Triticum_mon Treated | 20 Crusoe UnTreated | 30 Triticum_pol UnTreated | 40 Red_Lamas UnTreated |

Blocks 4

| | | | |
|---|---|---|---|
| 1 ASpeltoides UnTreated | 11 Cadenza Treated | 21 Victor UnTreated | 31 Malacca UnTreated |
| 2 Triticum_dico UnTreated | 12 Triticum_pol UnTreated | 22 Chidham Treated | 32 Hereward Treated |
| 3 Bulk_soil Treated | 13 Triticum_uran Treated | 23 Gallant Treated | 33 Bulk_soil UnTreated |
| 4 ASpeltoides Treated | 14 Triticum_macha UnTreated | 24 Crusoe UnTreated | 34 Avalon UnTreated |
| 5 Triticum_pol Treated | 15 Cadenza UnTreated | 25 Triticum_cart UnTreated | 35 Triticum_uratu Treated |
| 6 Red_Lamas UnTreated | 16 Crusoe Treated | 26 Malacca Treated | 36 Triticum_spe Treated |
| 7 Triticum_mon Treated | 17 Gallant UnTreated | 27 Triticum_uran UnTreated | 37 ATauschii Treated |
| 8 Victor Treated | 18 Triticum_macha Treated | 28 Triticum_mon UnTreated | 38 Triticum_uratu UnTreated |
| 9 Triticum_cart Treated | 19 Red_Lamas Treated | 29 Triticum_dico Treated | 39 Hereward UnTreated |
| 10 Triticum_spe UnTreated | 20 Chidham UnTreated | 30 ATauschii UnTreated | 40 Avalon Treated |

Glasshouse door

**Fig. S4.** Two-factor randomized block design of experimental plant layout with fertilization (UnTreated and Treated) and plant variety (*A. speltoides*, *A. tauschii*, Bulk soil, *T. aestivum* (cv.: Avalon, Cadenza, Chidham, Crusoe, Gallant, Hereward, Malacca, Red Lammas, Victor), *T. carthlicum*, *T. dicoccoides*, *T. macha*, *T. monococcum*, *T. polonicum*, *T. spelta*, *T. turanicum*, *T. urartu)* as factors. The design was split into four blocks based on greenhouse compartments. Each block consisted of 40 pots.

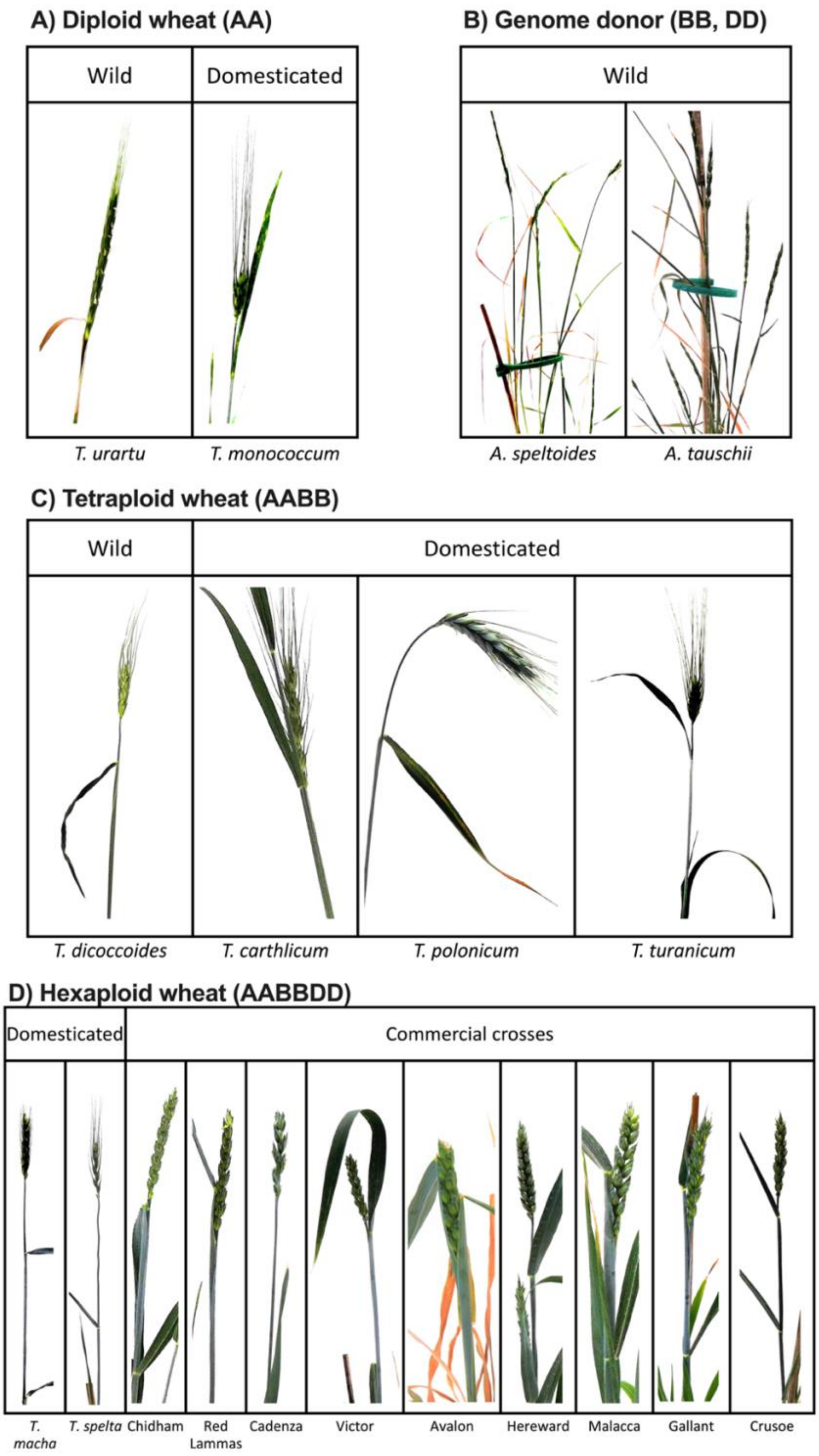


**Fig. S5.** Photos of wheat species used in this study, taken at flowering stage.

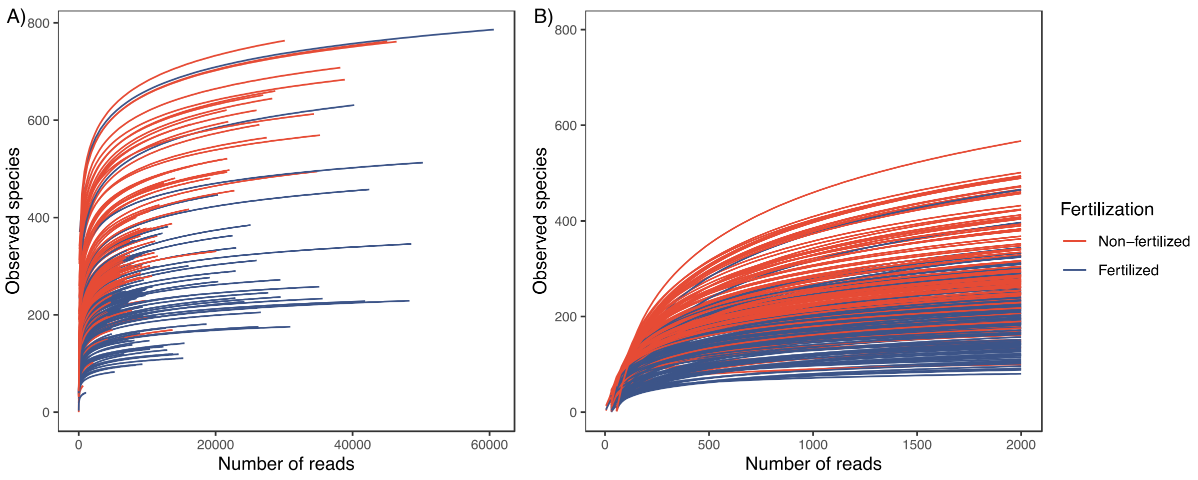


**Fig. S6.** Rarefaction curve analyses of bacterial species richness as a function of sequencing depth for (A) all samples and (B) samples at a cut-off of 2,000 reads as used for downstream alpha diversity analysis for culture-independent samples.

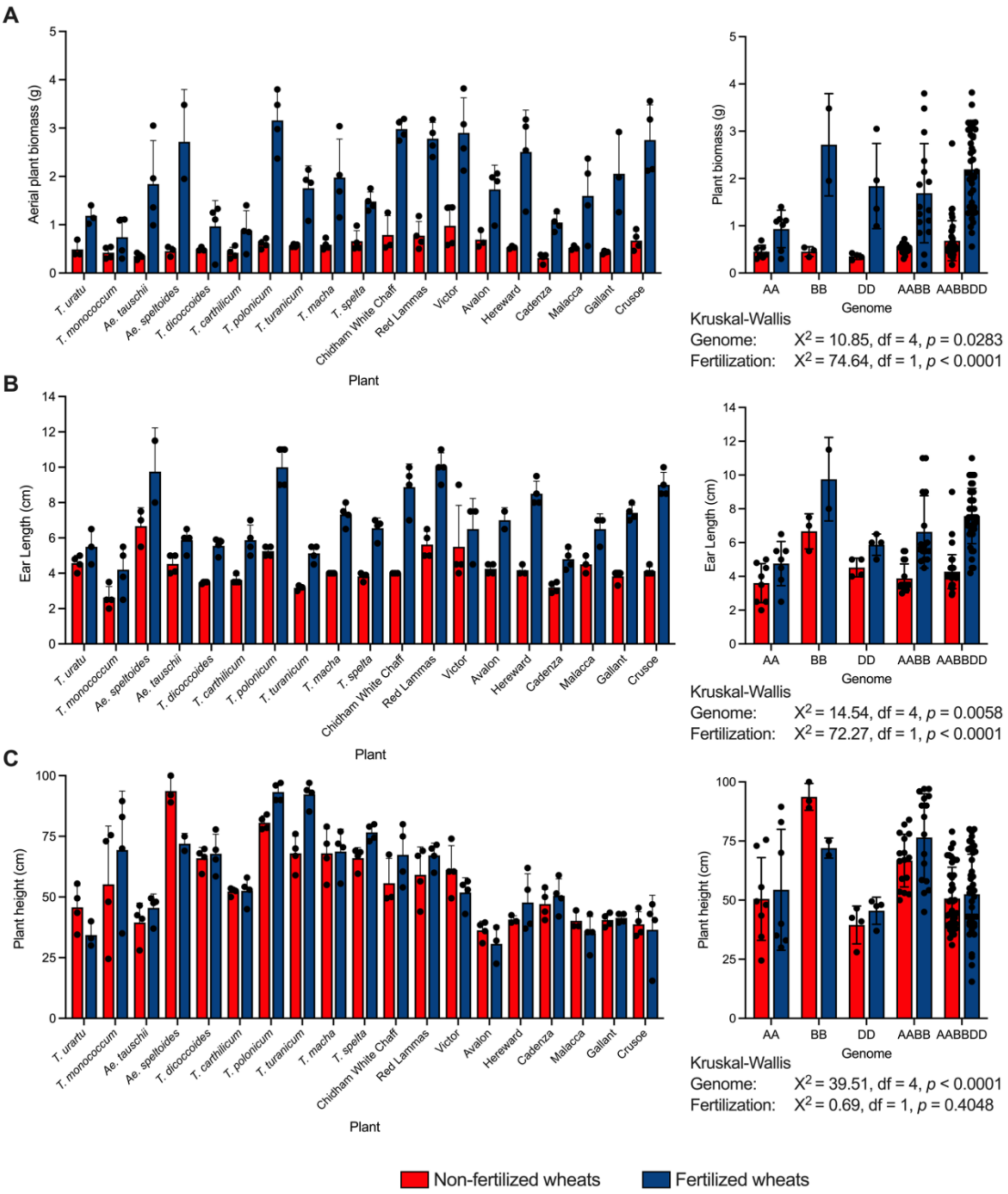


**Fig. S7.** Average dry plant biomass (A), ear length (B), and height (C) of wild wheat progenitors (AA, BB, DD) and allopolyploid (AABB, AABBDD) wheats grown in nutrient-depleted agricultural soil with and without fertilizer addition. Bars represent mean values for all 19 plant varieties (*Triticum* and *Aegilops*) from 4 biological replicates (left column) and for each wheat genome (right column) with individual samples shown as data points; error bars show the standard deviation. Statistical influences of genome level and fertilization were determined by Kruskal-Wallis tests. Plant height was measured from soil surface to head of longest stem, the longest ear of the plant was measured for ear length, and plant biomass was measured by dry foliar biomass.

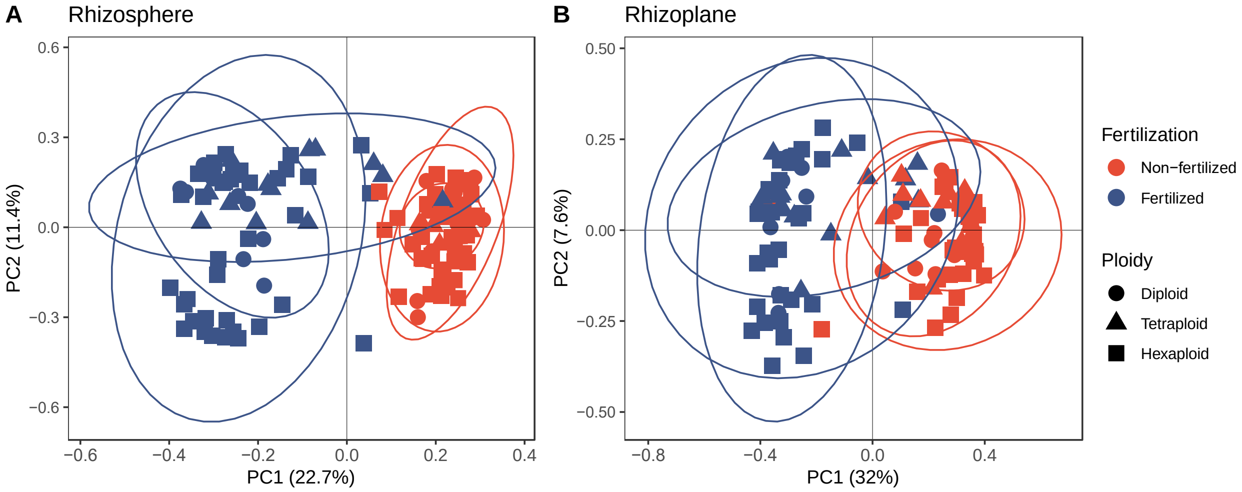


**Fig. S8.** Principal Coordinate Analysis (PCoA) plots of bacterial community based on Bray-Curtis distance in non-fertilized and fertilized rhizosphere (A) and rhizoplane (B) samples from diploid, tetraploid and hexaploid wheat varieties. The percentage shown in each axis corresponds to the proportion of variation explained.

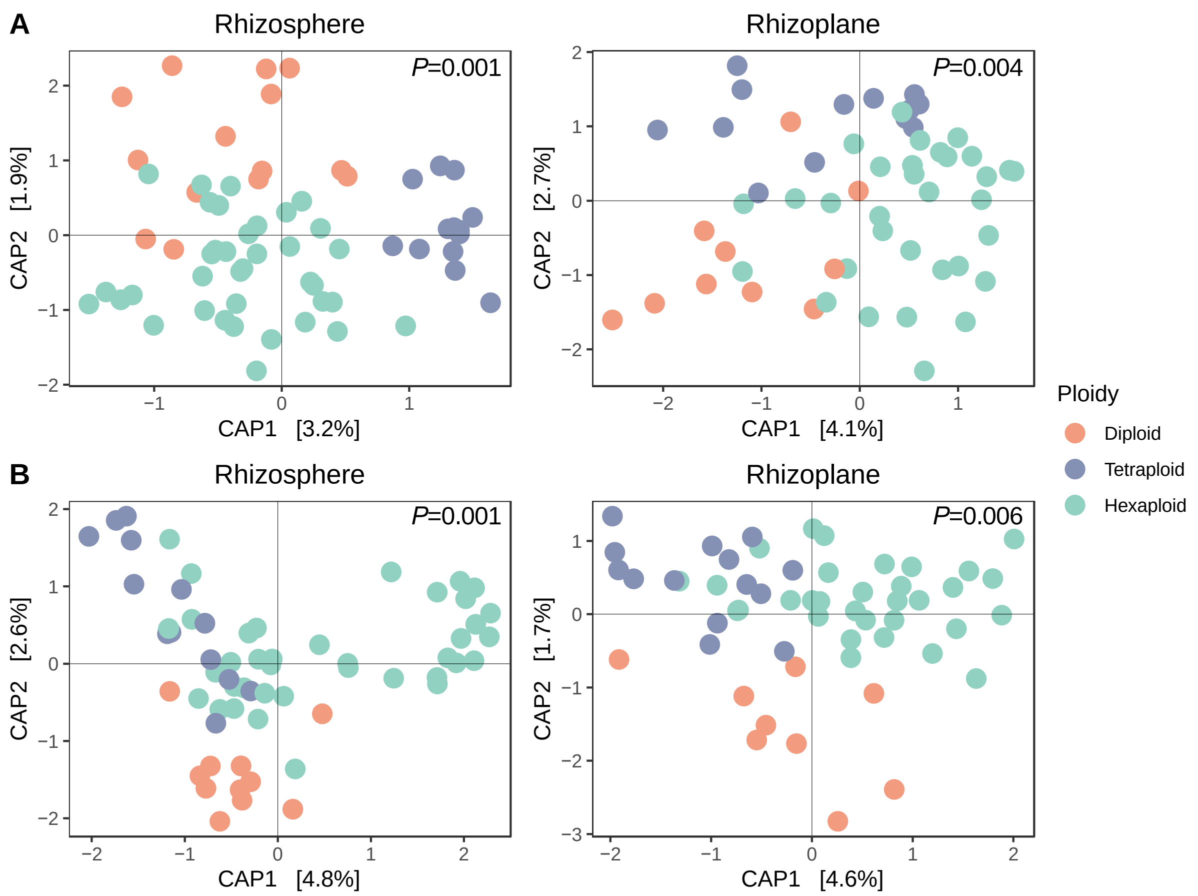


**Fig. S9.** Canonical Analysis of Principal coordinates (CAP) plots of bacterial community composition based on Bray-Curtis distance with ploidy level as the factor of constraint; *P* values are from permutation tests (ANOVA; capscale under a reduced model). The percentage shown in each axis corresponds to the proportion of variation explained.

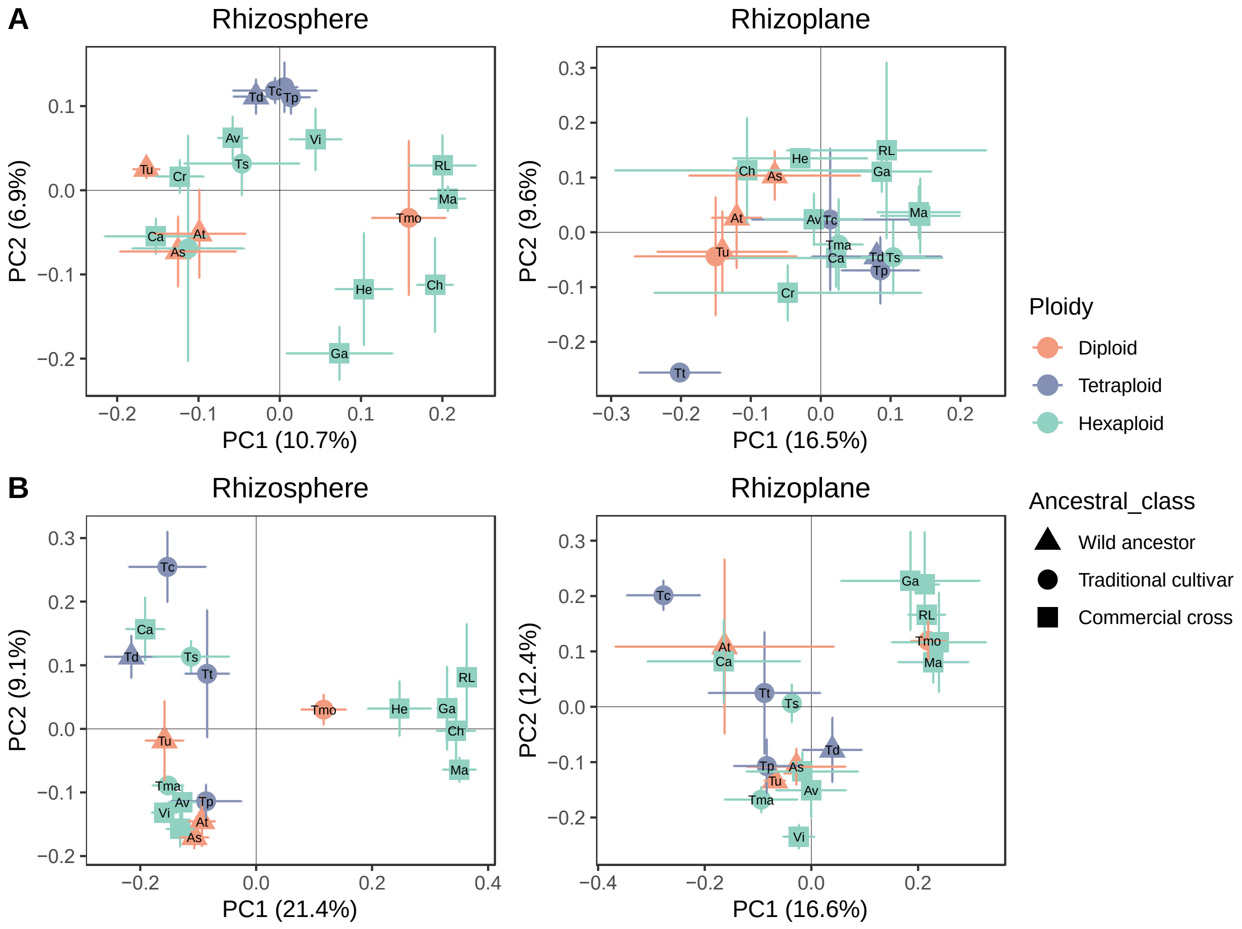


**Fig. S10.** PCoA plots of bacterial community based on Bray-Curtis distance in non-fertilized (A) and fertilized (B) rhizosphere and rhizoplane samples from diploid, tetraploid and hexaploid wheat varieties. Data points represent averaged (mean) location of all individual samples belonging to a given plant variety, while error bars represent standard deviation. The percentage shown in each axis corresponds to the proportion of variation explained. Text denotes plant species/variety abbreviations (see Table S1).

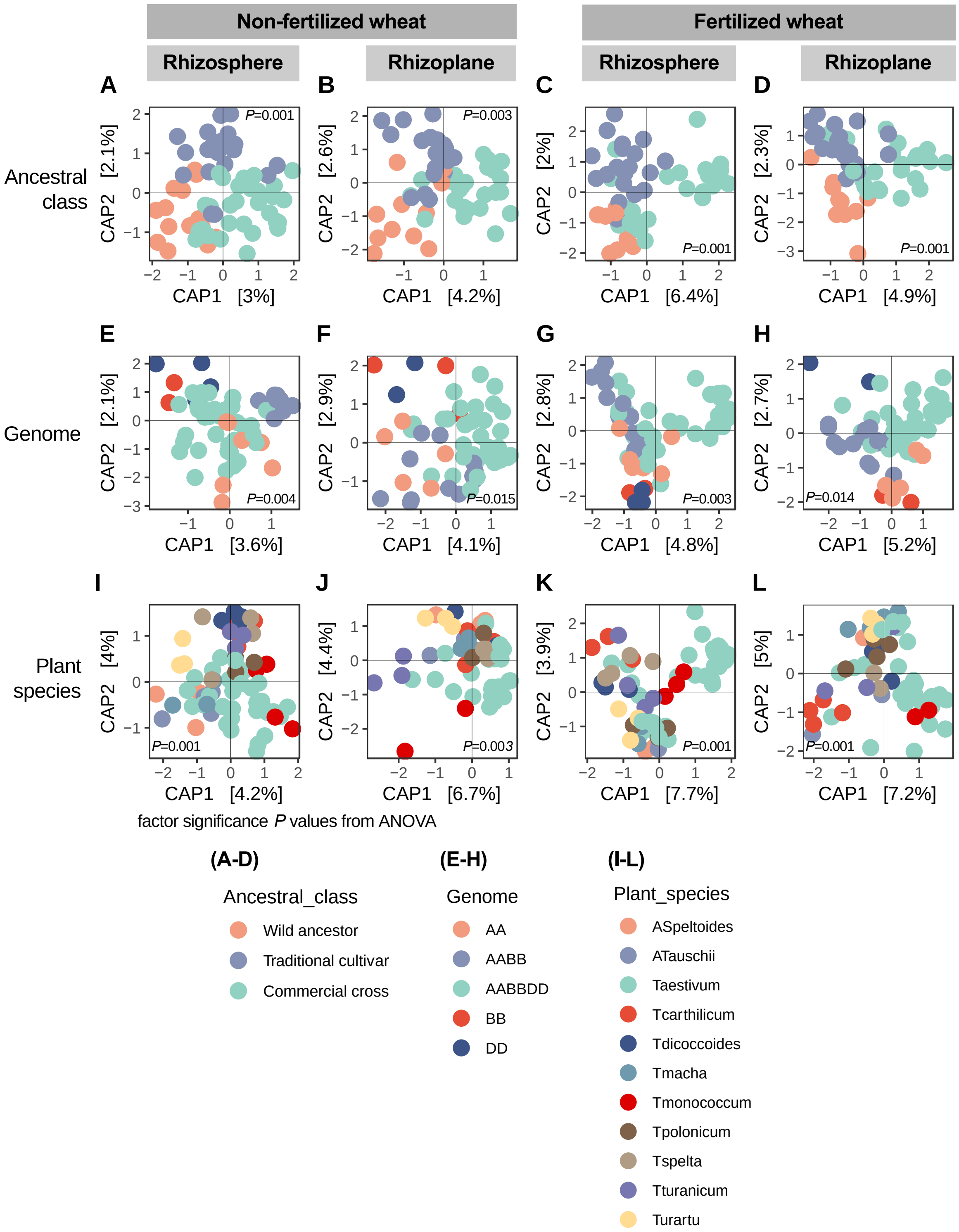


**Fig. S11.** CAP analysis of the bacterial rhizosphere (A, E, I) and rhizoplane (B, F, J) community from non-fertilized wheat (first two columns) and the bacterial rhizosphere (C, G, K) and rhizoplane (D, H, L) community for fertilized wheat (last two columns) using ancestral class (A-D), genome (E-H), and plant species (I-L) as the factors of constraint; *P* values are from permutation tests (ANOVA; capscale under a reduced model). The percentage shown in each axis corresponds to the proportion of variation explained.

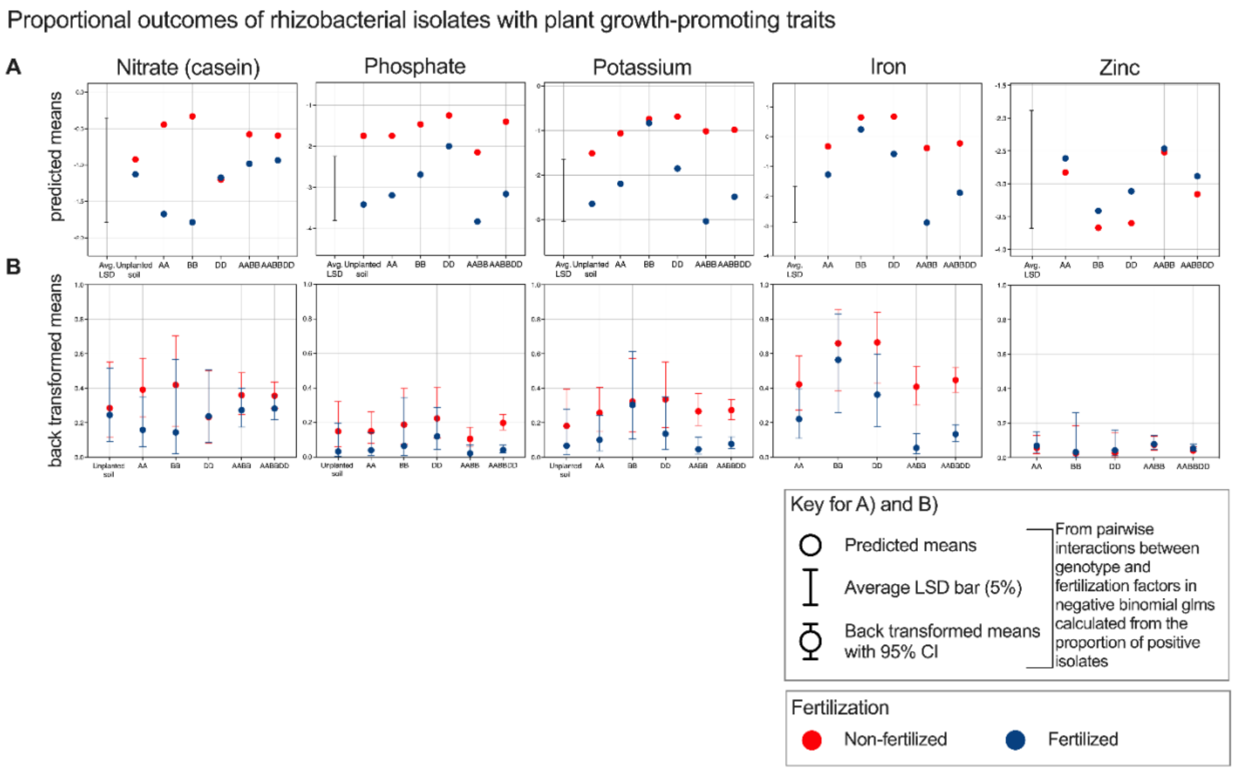


**Fig. S12.** Analysis of culturable bacterial abundances isolated from soil (unplanted and rhizoplane) samples collected from diploid wheat progenitors (AA, BB, DD), tetraploid (AABB) and hexaploid (AABBDD) wheats, grown with and without the addition of NPK fertilizer granules, as well as unplanted control pots. Plots of (A) predicted means with average least significant difference bars at 5% and (B) back transformed means with 95% confidence intervals (CI), calculated from negative binomial generalized linear models (glms) with genotype and fertilization as factors from the proportion of bacteria with corresponding nutrient acquisition traits.

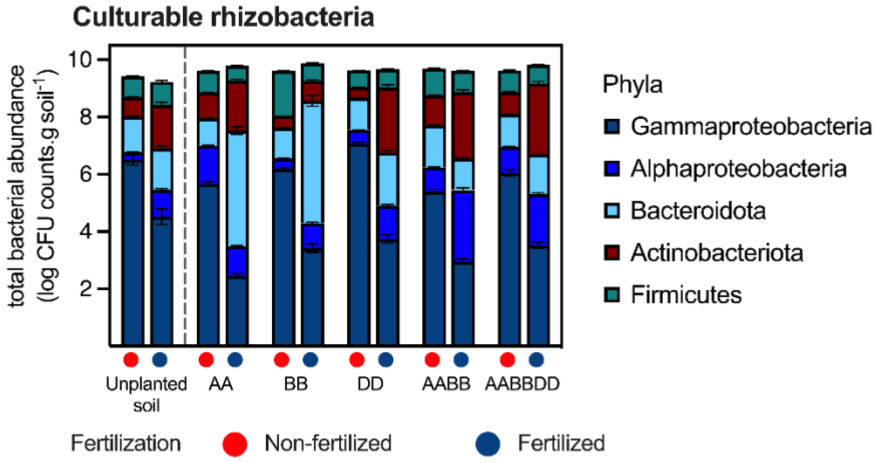


**Fig. S13.** Classification of phyla abundances in culturable bacterial communities isolated from soil (unplanted and rhizoplane) from diploid wheat progenitors (AA, BB, DD), tetraploid (AABB) and hexaploid (AABBDD) wheats, grown with and without the addition of NPK fertilizer granules, as well as unplanted control pots. Phyla percentages were calculated from 16S rRNA gene ASV counts (PGPR and non-PGPR) which were used to determine the absolute abundance of each phylum based on total bacterial abundance.

| | Plant | Line | Ancestral class | Ploidy | Genome | No. of biological reps. | |
|---|---|---|---|---|---|---|---|
| | | **Ancestral landraces** | | | | **No NPK** | **Plus NPK** |
| | | **Diploid wheat** | | | | | |
| 1 | *T. urartu* (Tu) | T1010038-PF-1 | wild ancestor | Diploid | AA | 4 | 3 |
| 2 | *T. monococcum* (Tm) | T1040005-PF-1 | traditional cultivar | Diploid | AA | 4 | 4 |
| | | **Tetraploid wheat** | | | | | |
| 3 | *T. dicoccoides* (Td) | T1060022-PF-3 | wild ancestor | Tetraploid | AABB | 4 | 4 |
| 4 | *T. carthlicum* (Tc) | T1100001-PF-1 | traditional cultivar | Tetraploid | AABB | 4 | 4 |
| 5 | *T. polonicum* (Tp) | T1090001-PF-1 | traditional cultivar | Tetraploid | AABB | 4 | 4 |
| 6 | *T. turanicum* (Tt) | T1110001-PF-1 | traditional cultivar | Tetraploid | AABB | 4 | 4 |
| | | **Hexaploid wheat** | | | | | |
| 7 | *T. macha* (Tma) | T1240001-PF-1 | traditional cultivar | Hexaploid | AABBDD | 4 | 4 |
| 8 | *T. spelta* (Ts) | T1220037-PF-1 | traditional cultivar | Hexaploid | AABBDD | 4 | 4 |
| | | **Commercial wheat** | | | | | |
| 9 | *T. aestivum* | Chidham White Chaff (Ch) | Not recorded | Hexaploid | AABBDD | 3 | 4 |
| 10 | *T. aestivum* | Red Lammas (RL) | Not recorded | Hexaploid | AABBDD | 4 | 4 |
| 11 | *T. aestivum* | Victor (Vi) | (Squarehead*Red King)*Talavera | Hexaploid | AABBDD | 4 | 4 |
| 12 | *T. aestivum* | Avalon (Av) | TJB 30/148* TL 365a/34/5 | Hexaploid | AABBDD | 4 | 4 |
| 13 | *T. aestivum* | Hereward (He) | Norman(sib)*Disponent | Hexaploid | AABBDD | 3 | 4 |
| 14 | *T. aestivum* | Cadenza (Ca) | Axona*Tonic | Hexaploid | AABBDD | 4 | 4 |
| 15 | *T. aestivum* | Malacca (Ma) | Riband*(Rendezvous)*Apostle | Hexaploid | AABBDD | 3 | 4 |
| 16 | *T. aestivum* | Gallant (Ga) | (Malacca*Charger)*Xi-19 | Hexaploid | AABBDD | 4 | 4 |
| 17 | *T. aestivum* | Crusoe (Cr) | Cordiale*Gulliver | Hexaploid | AABBDD | 4 | 4 |
| | | **Wild grass** | | | | | |
| | | **Genome donor** | | | | | |
| 18 | *A. speltoides* | T2140038-PF-1 | wild ancestor | Diploid | BB | 3 | 2 |
| 19 | *A. tauschii* | T2220012-PF-1 | wild ancestor | Diploid | DD | 4 | 4 |
| | unplanted | bulk soil control | | | | 4 | 4 |

**Table S1.** Wheat species chosen for the current study.

| Soil Parameter | Bare Fallow (*n=3)* |
|---|---|
| pH | 5.7 ± 0.1 |
| Moisture (%) | 11.1 ± 0.8 |
| Total C (%) | 0.9 ± 0.3 |
| Total N (%) | 0.1 ± 0.0 |
| C:N | 10.6 ± 0.3 |
| Inorganic C (%) | 0.0 ± 0.0 |
| $NO_3$ (kg dry soil) | 2.3 ± 0.0 |
| $NH_4$ (kg dry soil) | 0.6 ± 0.0 |
| Olsen P (kg dry soil) | 20.8 ± 1.1 |

**Table S2.** Soil properties from Woburn bare fallow soil sampled in April 2019.

| Non-fertilized wheat | | | | | | | | | | | |
|---|---|---|---|---|---|---|---|---|---|---|---|
| Rhizosphere | | | | | | Rhizoplane | | | | | |
| **Diploid** | | **Tetraploid** | | **Hexaploid** | | **Diploid** | | **Tetraploid** | | **Hexaploid** | |
| Acidobacteriota | 26.97% | Bacteroidota | 36.20% | Proteobacteria | 21.31% | Bacteroidota | 47.95% | Bacteroidota | 46.90% | Proteobacteria | 23.37% |
| Proteobacteria | 21.44% | Proteobacteria | 34.43% | Acidobacteriota | 19.09% | Proteobacteria | 19.59% | Proteobacteria | 23.64% | Bacteroidota | 22.64% |
| Bacteroidota | 11.54% | Actinobacteriota | 14.09% | Actinobacteriota | 9.78% | Chloroflexi | 6.94% | Patescibacteria | 17.09% | Patescibacteria | 22.24% |
| Actinobacteriota | 7.94% | Patescibacteria | 5.58% | Verrucomicrobiota | 9.13% | Actinobacteriota | 6.86% | Acidobacteriota | 5.95% | Acidobacteriota | 11.70% |
| Chloroflexi | 7.19% | Myxococcota | 3.35% | Cyanobacteria | 8.29% | Verrucomicrobiota | 5.07% | Actinobacteriota | 2.21% | Chloroflexi | 5.85% |
| Verrucomicrobiota | 4.92% | Acidobacteriota | 2.93% | Chloroflexi | 8.11% | Gemmatimonadota | 5.04% | Fibrobacterota | 0.96% | Fibrobacterota | 2.83% |
| Patescibacteria | 3.66% | Fibrobacterota | 1.39% | Bacteroidota | 7.36% | Patescibacteria | 3.04% | WPS_2 | 0.59% | Verrucomicrobiota | 2.74% |
| Planctomycetota | 3.13% | Bdellovibrionota | 1.21% | Patescibacteria | 3.85% | Acidobacteriota | 2.19% | Myxococcota | 0.59% | Firmicutes | 1.92% |
| Nitrospirota | 2.69% | Chloroflexi | 0.82% | Planctomycetota | 2.19% | Methylomirabilota | 1.69% | Bdellovibrionota | 0.57% | Desulfobacterota | 1.49% |
| Cyanobacteria | 2.52% | | | Nitrospirota | 2.17% | Desulfobacterota | 1.16% | Chloroflexi | 0.43% | Actinobacteriota | 1.15% |
| Desulfobacterota | 2.46% | | | FCPU426 | 1.67% | Cyanobacteria | 0.47% | Verrucomicrobiota | 0.30% | WPS_2 | 1.05% |
| Gemmatimonadota | 2.26% | | | Elusimicrobiota | 1.55% | | | Gemmatimonadota | 0.30% | Cyanobacteria | 0.90% |
| Methylomirabilota | 1.15% | | | Desulfobacterota | 1.53% | | | Cyanobacteria | 0.23% | Elusimicrobiota | 0.75% |
| FCPU426 | 1.09% | | | Dependentiae | 1.34% | | | Desulfobacterota | 0.12% | Latescibacterota | 0.41% |
| Latescibacterota | 1.05% | | | Gemmatimonadota | 0.92% | | | Firmicutes | 0.11% | RCP2_54 | 0.29% |
| | | | | Myxococcota | 0.91% | | | | | FCPU426 | 0.22% |
| | | | | Firmicutes | 0.80% | | | | | Planctomycetota | 0.17% |
| | | | | | | | | | | Myxococcota | 0.15% |
| | | | | | | | | | | Dependentiae | 0.15% |

| Fertilized wheat | | | | | | | | | | | |
|---|---|---|---|---|---|---|---|---|---|---|---|
| Rhizosphere | | | | | | Rhizoplane | | | | | |
| **Diploid** | | **Tetraploid** | | **Hexaploid** | | **Diploid** | | **Tetraploid** | | **Hexaploid** | |
| Proteobacteria | 20.83% | Proteobacteria | 43.69% | Proteobacteria | 45.96% | Bacteroidota | 64.98% | Proteobacteria | 39.28% | Proteobacteria | 39.34% |
| Actinobacteriota | 19.20% | Acidobacteriota | 11.50% | Actinobacteriota | 19.97% | Proteobacteria | 20.44% | Patescibacteria | 35.29% | Bacteroidota | 25.30% |
| Patescibacteria | 14.68% | Bacteroidota | 10.87% | Patescibacteria | 6.92% | Patescibacteria | 5.88% | Acidobacteriota | 7.83% | Patescibacteria | 19.47% |
| Acidobacteriota | 12.93% | Firmicutes | 9.37% | Acidobacteriota | 6.76% | Actinobacteriota | 5.03% | Bacteroidota | 4.96% | Actinobacteriota | 10.93% |
| Bacteroidota | 9.43% | Chloroflexi | 5.13% | WPS_2 | 4.21% | Firmicutes | 1.21% | Firmicutes | 4.70% | Firmicutes | 2.16% |
| Cyanobacteria | 7.59% | Actinobacteriota | 4.13% | Firmicutes | 3.98% | Chloroflexi | 0.89% | Actinobacteriota | 2.96% | Acidobacteriota | 1.00% |
| Chloroflexi | 6.60% | Verrucomicrobiota | 2.96% | Chloroflexi | 3.58% | Verrucomicrobiota | 0.47% | Chloroflexi | 1.76% | Chloroflexi | 0.93% |
| Firmicutes | 3.65% | Methylomirabilota | 1.96% | Bacteroidota | 2.92% | Gemmatimonadota | 0.31% | Nitrospirota | 0.77% | Cyanobacteria | 0.45% |
| Planctomycetota | 2.04% | Myxococcota | 1.74% | Cyanobacteria | 2.41% | Cyanobacteria | 0.23% | Latescibacterota | 0.58% | Verrucomicrobiota | 0.12% |
| Gemmatimonadota | 1.19% | Gemmatimonadota | 1.73% | Verrucomicrobiota | 0.90% | Bdellovibrionota | 0.16% | WPS_2 | 0.49% | Myxococcota | 0.11% |
| WPS_2 | 0.98% | RCP2_54 | 1.51% | Gemmatimonadota | 0.90% | Acidobacteriota | 0.15% | Verrucomicrobiota | 0.35% | Bdellovibrionota | 0.06% |
| Verrucomicrobiota | 0.87% | Patescibacteria | 1.25% | Planctomycetota | 0.78% | Myxococcota | 0.14% | Cyanobacteria | 0.31% | WPS_2 | 0.06% |
| | | Nitrospirota | 1.08% | Nitrospirota | 0.70% | Latescibacterota | 0.10% | Gemmatimonadota | 0.19% | Gemmatimonadota | 0.05% |
| | | Latescibacterota | 1.07% | | | | | Fibrobacterota | 0.16% | Planctomycetota | 0.04% |
| | | WPS_2 | 1.06% | | | | | Myxococcota | 0.14% | | |
| | | Cyanobacteria | 0.55% | | | | | Dependentiae | 0.13% | | |
| | | Desulfobacterota | 0.42% | | | | | Bdellovibrionota | 0.09% | | |

**Table S3.** Phyla from culture-independent amplicon sequence variant (ASV) datasets from non-fertilized rhizosphere, rhizoplane, and fertilized rhizosphere, rhizoplane wheat samples. The percentage of phyla abundances enriched in each ploidy group as detected by differential abundance analysis performed using DESeq2 (Wald test, individual $P$ values < 0.05, Benjamini-Hochberg procedure for multiple testing). Percentages for each phylum were calculated from the cumulative baseMean for ASVs (average of the normalized count values, divided by size factors, taken over all samples).

## References


1. Sarkar P, Stebbins GL. Morphological evidence concerning the origin of the B genome in wheat. Am. J. Bot. 1956;43(4):297-304.

2. Riley R, Unrau J, Chapman V. Evidence on the origin of the B genome of wheat. J. Hered. 1958;49(3):91-8.

3. Dvorák J, Zhang HB. Variation in repeated nucleotide sequences sheds light on the phylogeny of the wheat B and G genomes. PNAS. 1990;87(24):9640-44.

4. Maestra B, Naranjo T. Homoeologous relationships of *Aegilops speltoides* chromosomes to bread wheat. Theor. Appl. Genet. 1998;97:181-86.

5. Marcussen T, Sandve SR, Heier L, Spannagl M, Pfeifer M; International Wheat Genome Sequencing Consortium, *et al.* Ancient hybridizations among the ancestral genomes of bread wheat. Science. 2014;345(6194):1250092.

6. Dvořák J, Terlizzi P, Zhang HB, Resta P. The evolution of polyploid wheats: identification of the A genome donor species. Genome. 1993;36(1):21-31.

7. Hedden, P. The genes of the Green Revolution. Trends Genet. 2003;19(1):5-9.

8. Tkacz A, Pini F, Turner TR, Bestion E, Simmonds J, Howell P, *et al.* Agricultural selection of wheat has been shaped by plant-microbe interactions. Front. Microbiol. 2020;11:132.

9. Frazier WC, Rupp P. Studies on the proteolytic bacteria of milk i. a medium for the direct isolation of caseolytic milk bacteria. J. Bacteriol. 1928;16:57-63.

10. Pikovskaya RI. Mobilization of phosphorus in soil in connection with the vital activity of some microbial species. Microbiology. 1948;17:362-70.

11. Pratama D, Anas I, Suwarno. Ability of potassium-solubilising microbes to solubilise feldspar and their effects on sorghum growth. Malaysian J. Soil Sci. 2016;20:163-75.

12. Schwyn B, Neilands JB. Universal chemical assay for the detection and determination of siderophores. Anal. Biochem. 1987;160(1):47-56.

13. Louden BC, Haarmann D, Lynne AM. Use of blue agar CAS assay for siderophore detection. J. Microbiol. Biol. Educ. 2011;12(1):51-3.
