# Supplementary material for: Agricultural intensification reduces selection of putative plant growth-promoting rhizobacteria in wheat
Source: ISME J. 2024 Jul 11;18(1):wrae131. doi: 10.1093/ismejo/wrae131 (PMC11292143; doi:10.1093/ismejo/wrae131)
Supplement: Supplementary_Notes_wrae131 [file supplementary_notes_wrae131.pdf]

**Supplementary Notes**

# Agricultural intensification reduces selection of putative plant growth-promoting rhizobacteria in wheat

Tessa E. Reid[1,2], Vanessa N. Kavamura[1], Adriana Torres-Ballesteros[1], Monique E. Smith[1,3], Maïder Abadie[1,4+], Mark Pawlett[2], Ian M. Clark[1], Jim A. Harris[2] and Tim H. Mauchline[1*]

[1]Sustainable Soils and Crops, Rothamsted Research, Harpenden, Hertfordshire, AL5 2JQ, UK
[2]School of Water, Energy and Environment, Cranfield University, Cranfield, Bedfordshire MK43 0AL, UK
[3]Department of Ecology, Swedish University of Agricultural Sciences, Uppsala, SE-750 07, Sweden
[4]INRAE, UR1264 MycSA, CS2032, 33882 Villenave d'Ornon, France

+Current affiliation

*Corresponding author: Dr Tim H. Mauchline, Rothamsted Research, Harpenden, AL5 2JQ

tim.mauchline@rothamsted.ac.uk

**Contents**

**1. Extended Methods**

**Method S1** Seed sterilization and germination

**Method S2** Plant phenotypical traits

**Method S3** DNA extraction and PCR amplification

**Method S4** Bioinformatic analyses of 16S rRNA gene sequences

**Method S5** Statistical analysis

**2. Extended Results**

**Result S1** Plant phenotypical traits

**Result S2** Functional bioassay analysis of culturable isolates

**3. Extended Discussion**

**4. References**

# 1. Extended Methods

**Method S1** Seed sterilization and germination

Seeds were washed for 10 min in ethanol (70%), rinsed 5 times in sterile distilled water, soaked in 3% (w/v) sodium hypochlorite, rinsed 5 times again and then left to imbibe in sterile distilled water at 4°C overnight. Sterilized seeds were incubated for germination on pre-soaked (sterile distilled water) filter paper (autoclaved) in Petri dishes for three days in the dark at room temperature.

**Method S2** Plant phenotypical traits

Sampling took a total of four weeks for all wheat varieties to reach flowering stage. Height (from soil surface to head of longest stem) and ear length (length of longest ear) was measured, then soil was gently tipped from the pot onto a fresh polythene bag. Loose soil was discarded and non-rhizospheric soil carefully removed. After rhizosphere and rhizoplane samples had been taken, the remainder of the plant was dried (80°C, 24 h) and dry foliar plant biomass measured.

Plant height, ear length, and plant aerial biomass data were statistically analyzed with R Statistical Software v4.2.2 [1] in RStudio [2] using the kruskal.test and pairwise.wilcox.test function for non-normal data to investigate differences between fertilization and genome groups and pairwise interactions using the Benjamini & Hochberg procedure for multiple testing [3], respectively. Graphs were created in GraphPad Prism version 10.0.2 (171) for MacOS, GraphPad Software, Boston, Massachusetts USA, www.graphpad.com.

**Method S3** DNA extraction and PCR amplification

Genomic DNA was stored at -80°C. Extractions were performed according to the manufacturer's instructions but with the use of the FastPrep-24 5G machine (MP Biomedicals, Irvine, CA, USA) twice for 30 s at 5.5 m $s^{-1}$. DNA purity and concentrations were established on a NanoDrop 1000 spectrophotometer (Thermo Fisher Scientific, Wilmington, DE, United States), and a Qubit 2.0 Fluorimeter using the dsDNA HS assay kit (Thermo Fisher Scientific), respectively.

The V3-V4 region of bacterial 16S rRNA genes in samples were amplified using primers 314F (5'-CCTACGGGAGGCAGCAG-3') and 806R (5'-GGACTACHVGGGTWTCTAAT-3') [4]. All PCR reactions were conducted with the

following setup: 15 µl 2x KAPA HiFi HotStart ReadyMix PCR (Roche, Basel, Switzerland), 3 µl of each 0.5 µM forward and reverse primer indexed solutions, 9 µl PCR grade water, and 3 µl DNA template (150 ng). PCR amplifications were performed on a C1000 Touch Thermal Cycler (Bio-Rad Laboratories, Hercules, CA, USA) under the following conditions: 95 °C for 3 min; 30 cycles of 95 °C for 20 s, 55 °C for 15 s, 72 °C for 20 s; followed by 72 °C for 1 min. We included two negative control samples (blank DNA extractions) and two mock community samples. Indexed PCR products were cleaned with Agencourt AMPure XP beads (Beckman Coulter, Brea, CA, USA) and normalized to equimolar concentration prior to being pooled. The final pool was quantified, and quality assessed using SYBR green quantitative PCR assay with primers specific to the Illumina adapters (Kappa) and diluted to 12 pM. The library was spiked with PhiX control V3 library (5% v/v, 12.5 pM).

**Method S4** Bioinformatic analyses of 16S rRNA gene sequences

The raw 16S rRNA gene amplicon sequences from the culture-independent data set and the culture-dependent data set were processed separately using QIIME (Quantitative Insights into Microbial Ecology) [5] 2 v2019.7. Paired-end reads were imported into QIIME 2 using the q2-demux plugin for demultiplexing. Culture-independent sequences were merged using the vsearch [6] merge_pairs function which produced 6 million reads from 302 samples (median 16,000 reads per samples) (298 samples, 2 negative controls, and 2 mock community samples). Merged sequences were then denoised using DADA2 [7] v1.18.0 (via q2-dada2) where sequences were processed into amplicon sequence variants (ASVs) using default parameters. Culture-dependent sequences were merged and denoised using DADA2 for paired-end reads which produced 20 million reads from 196 samples (median 110,861 reads per sample) (193 samples, 1 negative control, and 2 mock community samples) to produce ASVs. Samples with less than 100 reads were removed (n=17 from culture-independent dataset) and ASVs with less than 10 observations in the entire data set were excluded. Bacterial taxonomy was assigned to the genus level using the q2-feature-classifier [8] classify-consensus-vsearch [6] against the SILVA database [9, 10] (version 138). ASVs that were not classified as bacteria at kingdom level were removed and the resulting ASVs were aligned with mafft [11] and used to build a phylogenetic tree with fasttree [12] using the q2-align-to-tree-mafft-fasttree function via q2-phylogeny. The resulting ASV tables, taxonomy assignments, and phylogenetic trees were exported for downstream analysis.

**Method S5** Statistical analysis

Statistical analyses were performed in R v4.2.2 [1] using RStudio [2] unless otherwise stated. The predictmeans package [13] was used to make pairwise comparisons from negative binomial generalized linear models (glm) based on fertilization and genome interaction ($P < 0.05$). This generated predicted means for each group with average least significant difference (LDS) bars at 5% and back transformed means with 95% confidence internals from the negative binomial glm, as well as pairwise comparisons. The predicted mean plots were used to interpret differences and trends between samples based on the average LSD bar; the back transformed means showed the true proportional value.

For all data tested by two-way analysis of variance (ANOVA) tests, normality was confirmed by quantile-quantile normality plots and Shapiro-Wilk tests [14] and homogeneity of variances was confirmed by residuals versus fits plots and Spearman's test for heteroscedasticity. All two-way ANOVA tests used type III sums of squares. For statistical analysis of absolute abundances of rhizobacteria, outliers were removed using the ROUT method [15] in Prism10.

Permutational multivariate analysis of variance (PERMANOVA) was used to detect significant changes in the bacterial community structure using pseudo-F value as a proxy for the strength of an individual factor (fertilization, soil, ancestral class, ploidy, genome, and plant species) on the bacterial community composition. Two PERMANOVA types were used: (1) permutation test under a reduced model, sum of squares type I (sequential) with 9,999 permutations using fixed multifactorial design; (2) multifactorial, nested design PERMANOVA (type I) where plant species were nested in genome which were nested in ploidy.

The R package DESeq2 v1.36.0 [17] was used to identify differentially abundant ASVs enriched in: 1) non-fertilized vs. fertilized wheats in the culture-independent dataset; 2) diploid, tetraploid, vs. hexaploid wheats in the culture-independent dataset; 3) diploid, tetraploid, vs. hexaploid wheats in the culture-dependent dataset, using the Wald test with a local fit type and adjusted *P* values to correct for multiple testing using the Benjamini-Hochberg procedure [3] at a significance level of < 0.0001 for (1) and < 0.05 for (2) and (3). The resulting data were used to produce volcano plots with the R package ggplot2 and ternary plots using the methods reported in Bulgarelli et al. (2012) [18], to visualize ASVs preferentially associated with fertilization or ploidy level, respectively. For phyla abundances, the cumulative baseMean values, which is the average of the normalized ASV count values, divided by size factors, taken

over all samples, from the DESeq2 results were plotted in Prism 10. The same data was used to plot genus abundances for culture-dependent ASVs classified to genus level, also in Prism 10.

## 2. Extended Results

**Result S1** Plant phenotypical traits

Fertilization had the biggest impact on plant biomass and ear length (Kruskal-Wallis: $\chi^2$ = 74.64, df (degrees of freedom) = 1, $P < 0.0001$ and $\chi^2$ = 72.3, df = 1, $P < 0.0001$, respectively) compared to genome ($\chi^2$ = 10.9, df = 4, $P$ = 0.0283 and $\chi^2$ = 14.54, df = 4, $P$ = 0.0058, respectively) (Fig. S7A and S7B). Wheat genome significantly influenced plant height ($\chi^2$ = 39.5, df = 4, $P < 0.0001$) with genotypes BB (85.0 ± 12.7 cm, $n$ = 5) and AABB (71.6 ± 15.8 cm, $n$=32) being taller than genotypes: AA (52.3 ± 20.9 cm, $n$ = 15), AABBDD (51.6 ± 15.2 cm, $n$ = 84), and DD (42.5 ± 7.2 cm, $n$ = 8) (Wilcoxon rank sum test with Benjamini-Hochberg correction, $P < 0.05$) (Fig. S7C). Fertilization had no effect on plant height ($\chi^2$ = 0.7, df = 1, $P$ = 0.4048) (Fig. S7C).

**Result S2** Functional bioassay analysis of culturable isolates

In general, rhizoplane bacteria that tested positive for solubilization of plant macronutrients: organic N (casein), inorganic phosphate ($Ca_3(PO_4)_2$), potassium (potash feldspar), and plant micronutrient: iron ($FeCl_3 \cdot 6H_2O$) had a statistically higher relative abundance in isolate libraries cultured from non-fertilized compared to fertilized wheat samples, from all wheat genotypes (analysis of deviance: $P < 0.05$) (Fig. S12) (Data S5). The general trend for P, K, and Fe -solubilizing bacterial isolates was an increase in abundance from unplanted soil, AA, BB, and DD wheat rhizoplane and a decrease in abundance from AABB and AABBDD rhizoplane samples with a significantly lower proportion of isolates cultured from AABB and AABBDD wheats grown with fertilizer (Fig. S12). In contrast, there were no statistical differences in abundances of zinc (ZnO)-solubilizing bacteria caused by fertilization or by genotype (Zinc in Fig. S12) (Data S5).

## 3. Extended Discussion

There was no clear difference between ploidy levels in terms of rhizobacterial species richness, diversity, evenness, and phylogenetic diversity for a given fertilization regime. This has been observed previously with alpha diversity indices related to domestication [19]. This was also the case for prokaryotic diversity in maize rhizosphere and rhizoplane microbial communities with there being no difference between teosinte, inbred, and modern maize [20], as well as for the Shannon and Simpson index in wild versus domesticated rice [21]. A study did show that polyploid wheats harboured greater rhizosphere bacterial diversity than diploid wheats but that this trend was more pronounced in field plots compared to pot experiments and that results varied based on field sampling year [22]. In fact, our results showed there to be higher phylogenetic diversity in the rhizoplane of hexaploid wheats compared to diploid wheats under non-fertilized conditions (Data S1) which does support these findings. Another study also showed a similar result between diploid (*T. monococcum*), tetraploid (*T. turgidum*), and hexaploid (*T. aestivum* varieties and *T. spelta*) wheat cultivars; there was no difference between rhizobacterial richness and diversity in wheat sampled at 21 weeks post germination when the first side shoot was visible however, when sampling at an earlier growth stage (emergence of first leaf 10 weeks post germination) alpha diversity was higher in hexaploid when compared with diploid and tetraploid wheats [23]. Additionally, root bacterial richness and diversity was significantly higher in wild wheat roots compared to land and inbred races of wheat at two-weeks post germination [24]. This suggests that the initial phase of microbial colonization is a better stage to assess differences in rhizobacterial alpha diversity between wheats when based on ploidy status. This would explain the similar rhizobacterial alpha diversity results in this study of wheats sampled at late-stage development (flowering).

## 4. References


1. Team RC. R: A language and environment for statistical computing. R Foundation for Statistical Computing, Vienna, Austria. https://www.R-project.org/ 2022.

2. Team R. RStudio: Integrated development environment for R. RStudio, Inc., Boston, MA. http://www.rstudio.com/ 2022.

3. Benjamini Y, Hochberg Y. Controlling the false discovery rate: a practical and powerful approach to multiple testing. J. R. Stat. Soc. Series B Methodol. 1995;57:289-300.

4. Klindworth A, Pruesse E, Schweer T, Peplies J, Quast C, Horn M, *et al.* Evaluation of general 16S ribosomal RNA gene PCR primers for classical and next-generation sequencing-based diversity studies. Nucleic Acids Res. 2013;41(1):e1.

5. Bolyen E, Rideout JR, Dillon MR, Bokulich NA, Abnet CC, Al-Ghalith GA*, et al.* Reproducible, interactive, scalable and extensible microbiome data science using QIIME 2. Nat. Biotechnol. 2019;37:852-57.

6. Rognes T, Flouri T, Nichols B, Quince C, Mahé F. VSEARCH: a versatile open source tool for metagenomics. PeerJ. 2016;4:e2584.

7. Callahan BJ, McMurdie PJ, Rosen MJ, Han AW, Johnson AJ, Holmes SP. DADA2: High-resolution sample inference from Illumina amplicon data. Nat. Methods. 2016;13:581-3.

8. Bokulich NA, Kaehler BD, Rideout JR, Dillon M, Bolyen E, Knight R*, et al.* Optimizing taxonomic classification of marker-gene amplicon sequences with QIIME 2's q2-feature-classifier plugin. Microbiome. 2018;6:90.

9. Quast C, Pruesse E, Yilmaz P, Gerken J, Schweer T, Yarza P*, et al.* The SILVA ribosomal RNA gene database project: improved data processing and web-based tools. Nucleic Acids Res. 2012;41(D1):D590-96.

10. Yilmaz P, Parfrey LW, Yarza P, Gerken J, Pruesse E, Quast C*, et al.* The SILVA and "All-species Living Tree Project (LTP)" taxonomic frameworks. Nucleic Acids Res. 2013;42(D1):D643-48.

11. Katoh K, Misawa K, Kuma K, Miyata T.. MAFFT: a novel method for rapid multiple sequence alignment based on fast Fourier transform. Nucleic Acids Res. 2002;30(14):3059-66.

12. Price MN, Dehal PS, Arkin AP. FastTree 2 – approximately maximum-likelihood trees for large alignments. PLOS One. 2010;5(3):e9490.

13. Luo D, Ganesh S, Koolaard J. predictmeans: predicted means for linear and semi parametric models. R package version 1.0.8. 2022. https://CRAN.R-project.org/package=predictmeans.

14. Shapiro SS, Wilk MB. An analysis of variance test for normality (complete samples). Biometrika. 1965;52(3-4):591-611.

15. Motulsky HJ, Brown RE. Detecting outliers when fitting data with nonlinear regression – a new method based on robust nonlinear regression and the false discovery rate. BMC Bioinformatics. 2006;7:123.

16. Oksanen J, Simpson GL, Blanchet FG, Kindt R, Legendre P, Minchin PR, *et al.* vegan: community ecology package. R package version 2.6-4. 2022. https://CRAN.R-project.org/package=vegan.

17. Love MI, Huber W, Anders S. Moderated estimation of fold change and dispersion for RNA-seq data with DESeq2. Genome Biol. 2014;15(12):550.

18. Bulgarelli D, Rott M, Schlaeppi K, Ver Loren van Themaat E, Ahmadinejad N, Assenza F, et al. Revealing structure and assembly cues for Arabidopsis root-inhabiting bacterial microbiota. Nature. 2012;488(7409):91-5.

19. Spor A, Hassani MA, Sesiz U, Künzel S, Dagan T, Özkan H, *et al.* Domestication-driven changes in plant traits associated with changes in the assembly of the rhizosphere microbiota in tetraploid wheat. Sci. Rep. 2020;10:12234.

20. Brisson VL, Schmidt JE, Northen TR, Vogel JP, Gaudin ACM. Impacts of maize domestication and breeding on rhizosphere microbial community recruitment from a nutrient depleted agricultural soil. Sci. Rep. 2019;9:15611.

21. Chang J, Sun Y, Tian L, Ji L, Luo S, Nasir F, *et al.* The structure of rhizosphere fungal communities of wild and domesticated rice: changes in diversity and co-occurrence patterns. Front. Microbiol. 2021;12:610823.

22. Wipf HML, Coleman-Derr D. Evaluating domestication and ploidy effects on the assembly of the wheat bacterial microbiome. PLOS One. 2021;16(3):e0248030.

23. Kinnunen-Grubb M, Sapkota R, Vignola M, Nunes IM, Nicolaisen M. Breeding selection imposed a differential selective pressure on the wheat root-associated microbiome. FEMS Microbiol. Ecol. 2020;96(11):fiaa196.

24. Özkurt E, Hassani MA, Sesiz U, Künzel S, Dagan T, Özkan H, *et al.* Seed-derived microbial colonization of wild emmer and domesticated bread wheat (*Triticum dicoccoides* and *T. aestivum*) seedlings shows pronounced differences in overall diversity and composition. mBio. 2020;11(6):e02637-20.
